# Early Kinetics of the HLA Class I-Associated Peptidome of MVA.HIVconsv-Infected Cells

**DOI:** 10.1128/JVI.03627-14

**Published:** 2015-03-25

**Authors:** Nicola Ternette, Peter D. Block, Álvaro Sánchez-Bernabéu, Nicola Borthwick, Elisa Pappalardo, Sultan Abdul-Jawad, Beatrice Ondondo, Philip D. Charles, Lucy Dorrell, Benedikt M. Kessler, Tomáš Hanke

**Affiliations:** a The Jenner Institute, Nuffield Department of Medicine, University of Oxford, Oxford, United Kingdom; b Target Discovery Institute, Nuffield Department of Medicine, University of Oxford, Oxford, United Kingdom; c Universitat de Barcelona, Barcelona, Spain; d Sir William Dunn School of Pathology, University of Oxford, Oxford, United Kingdom

## Abstract

Cytotoxic T cells substantially contribute to the control of intracellular pathogens such as human immunodeficiency virus type 1 (HIV-1). Here, we evaluated the immunopeptidome of Jurkat cells infected with the vaccine candidate MVA.HIVconsv, which delivers HIV-1 conserved antigenic regions by using modified vaccinia virus Ankara (MVA). We employed liquid chromatography-tandem mass spectrometry (LC-MS/MS) to identify 6,358 unique peptides associated with the class I human leukocyte antigen (HLA), of which 98 peptides were derived from the MVA vector and 7 were derived from the HIVconsv immunogen. Human vaccine recipients responded to the peptide sequences identified by LC-MS/MS. Peptides derived from the conserved HIV-1 regions were readily detected as early as 1.5 h after MVA.HIVconsv infection. Four of the seven conserved peptides were monitored between 0 and 3.5 h of infection by using quantitative mass spectrometry (Q-MS), and their abundance in HLA class I associations reflected levels of the whole HIVconsv protein in the cell. While immunopeptides delivered by the incoming MVA vector proteins could be detected, all early HIVconsv-derived immunopeptides were likely synthesized *de novo*. MVA.HIVconsv infection generally altered the composition of HLA class I-associated human (self) peptides, but these changes corresponded only partially to changes in the whole cell host protein abundance.

**IMPORTANCE** The vast changes in cellular antigen presentation after infection of cells with a vectored vaccine, as shown here for MVA.HIVconsv, highlight the complexity of factors that need to be considered for efficient antigen delivery and presentation. Identification and quantitation of HLA class I-associated peptides by Q-MS will not only find broad application in T-cell epitope discovery but also inform vaccine design and allow evaluation of efficient epitope presentation using different delivery strategies.

## INTRODUCTION

T cells recognize foreign peptide antigens that are bound to major histocompatibility complexes (MHCs), which are transported and presented on the surface of the cell. The current paradigm states that peptides destined for MHC class I presentation are typically generated from proteins that were directly synthesized in or reached the cell cytoplasm, while peptides associated with MHC class II are typically produced from proteins engulfed in the endosomal or lysosomal compartments (1, 2).

Presentation of foreign peptides by MHC molecules is important for the priming of T-cell responses by professional antigen-presenting cells and for the recognition and destruction of invading infected target cells. Following infection, a hierarchy of T-cell responses (and the corresponding cognate epitopes) is established. This response is determined first by not very well understood processes governing the amounts and time of each foreign peptide presented on the cell surface and second by the T-cell receptor repertoire of the host genome, which is defined by the numbers of naive T cells and their subsequent shaping by thymic selection (3–5). In order to reliably reflect the actual protein content of a cell at any given moment, the repertoire of peptides presented on the cell surface needs to be revised and reconstituted constantly. Therefore, the pool of MHC-associated peptides, or immunopeptidome, is highly dynamic. Monitoring of MHC-peptide complexes is carried out primarily by employing functional T-cell assays (6). Mass spectrometry (MS)-based identification of MHC-associated peptide antigens has become an increasingly feasible, less biased alternative (7–9). More recent technical advances allowed the profiling of several thousand peptide epitopes of the immunopeptidome in one experiment (10, 11).

CD8^+^ effector T cells play a key role in the control of human immunodeficiency virus type 1 (HIV-1) replication during both the acute and chronic phases of infection (12). The first dominant CD8^+^ T-cell responses generated at the onset of HIV-1 infection recognize peptides from the most variable regions of HIV-1 proteins. Under selective immune pressure, these epitopes easily change and render mutant HIV-1 unrecognizable by the mounted responses. Such mutant viruses rapidly overgrow their suppressed parental strains and replace them. This represents a major challenge for the development of effective vaccines (12–14). To tackle HIV-1 escape, we designed the vaccine immunogen HIVconsv (15), which focuses T cells on the most conserved regions of the HIV-1 proteome common to most circulating strains, in which mutations are often detrimental to HIV-1 fitness (16–19). Such conserved epitopes are typically subdominant in immune responses to natural infection; however, demonstration of an efficient and timely presentation of these epitopes on the cell surface during vaccination may induce dominant responses and therefore would support the conserved-region vaccine strategy.

The HIVconsv gene has been inserted into many vaccine vectors mainly for administration in heterologous prime-boost regimens (15, 20–24). One such vector on the forefront of vaccine development for many infectious diseases is modified vaccinia virus Ankara (MVA) (25, 26). Recombinant MVA primes transgene-specific T cells weakly but delivers a very strong boost to existing responses (27–31). Indeed, HIVconsv delivered to healthy human volunteers using combinations of plasmid DNA, simian (chimpanzee) adenovirus, and MVA induced uniquely high frequencies of HIVconsv-specific T cells (27).

Here, we report the identification of 6,358 unique peptides associated with class I human lymphocyte antigen (HLA), the human MHC, following infection of human cells with the candidate HIV-1 vaccine MVA.HIVconsv using liquid chromatography-tandem mass spectrometry (LC-MS/MS). Of these peptides, 98 were derived from the MVA vector, and 7 were derived from the HIVconsv immunogen. Using quantitative mass spectrometry (Q-MS), we monitored the early kinetics of 4 HIVconsv-derived epitopes and correlated their abundances to the amounts of HIVconsv protein in the cytoplasm. We also demonstrate that epitopes derived from incoming virions can be directly presented by HLA class I complexes. These results are discussed in the context of HIV-1 vaccine design and antigen presentation.

## MATERIALS AND METHODS

### MVA.HIVconsv vaccine propagation and titration.

MVA.HIVconsv was constructed previously (15). MVA.HIVconsv was propagated in primary chicken embryonic fibroblasts (CEFs) maintained in Dulbecco's modified Eagle medium (DMEM) supplemented with 2% fetal calf serum (FCS) (DMEM2). Cells were infected at a multiplicity of infection (MOI) of 0.1, harvested 3 days later, and homogenized in 10 mM Tris (pH 9.0) by 20 strokes in a Potter-Elvehjem glass homogenizer. Cell lysates were layered on a 30% sucrose cushion and ultracentrifuged for 45 min at 20,000 × *g*. Virus pellets were suspended in 10 mM Tris (pH 9.0) and stored at −80°C. The titers of MVA.HIVconsv viral stocks were determined by infection of CEF cells in a 6-well format for 3 days in DMEM2 at serial dilutions of between 10^−6^ and 10^−11^. Infected cells were visualized by using 5-bromo-4-chloro-3-indolyl-β-d-galactopyranoside (X-Gal), a substrate of β-galactosidase which is expressed in MVA.HIVconsv-infected cells. Blue cells were counted, and the numbers of infectious particles in the original stock were calculated, assuming one infectious particle per infected cell.

### Jurkat cell infection and stable isotope labeling by amino acids in cell culture (SILAC).

Jurkat cells were cultured in either RPMI 1640 medium supplemented with 10% FCS (RPMI10) or RPMI10 supplemented with heavy arginine (R10) and heavy lysine (K4 or K8) for a minimum of 10 passages. Cells were seeded the day before infection at a density of 5 × 10^5^ cells/ml. An equivalent of 10 infectious particles per cell (MOI of 10) was added to RPMI 1640 medium supplemented with 2% FCS with or without heavy arginine (R10) and heavy lysine (K4 or K8) at a concentration of 10^7^ cells/ml. At the indicated time points postinfection, cells were pelleted at 300 × *g* for 5 min, washed once with phosphate-buffered saline (PBS), and frozen at −20°C until processing for either HLA-associated peptide purification or tryptic digestion of cell lysates.

### Immunofluorescence staining.

HeLa cells were infected with MVA.HIVconsv at an MOI of 5 for 2 h at 37°C in 5% CO_2_ in serum-free medium. Following infections, cells were washed with PBS, incubated in complete medium for 24 h, washed twice with ice-cold PBS, and fixed with a 10% formalin solution, neutral buffered containing 4% formaldehyde (Sigma), for 10 min on ice and then for 20 min at room temperature. Cells were then washed with PBS 3 times, permeabilized with 0.2% Triton X-100 (TX-100) (Sigma) in PBS for 5 min, washed, blocked with 1% bovine serum albumin (BSA) in PBS for 30 min, incubated for 3 h with a 1:100 dilution of the Alexa Fluor 488-conjugated primary mouse anti-Pk monoclonal antibody (MAb) (AbD Serotec), washed with PBS 3 times for 15 min with shaking, mounted onto microscope slides with Vectashield 4′,6-diamidino-2-phenylindole (DAPI) nuclear stain mounting medium (Vector Laboratories), and examined on a fluorescence microscope (DMI 3000B; Leica).

### Preparation of W6/32-conjugated immunoresin.

One milliliter of protein A-Sepharose beads (GE) was washed in a solution containing 50 mM borate and 50 mM KCl (pH 8.0) and incubated with 2 to 5 mg of W6/32 antibody with mild rotation for 1 h. The beads were washed in 0.2 M triethanolamine (pH 8.2), and the bound antibody was cross-linked by using 40 mM dimethyl pimelimidate dihydrochloride (DMP) (Sigma) (pH 8.3) for 1 h at room temperature. The reaction was quenched by the addition of ice-cold 0.2 M Tris buffer (pH 8.0) to the mixture. Unbound antibody was removed by using 0.1 M citrate (pH 3.0), and the column was equilibrated in 50 mM Tris (pH 8.0) for further use.

### HLA class I immunoprecipitation.

All steps were carried out at temperatures below 4°C. Briefly, cell pellets of 10^9^ cells per sample were lysed by using 10 ml lysis buffer (1% IGEPAL 630, 300 mM NaCl, 100 mM Tris [pH 8.0]) and homogenized by mild sonication. Lysates were cleared by two subsequent centrifugation steps, one at 300 × *g* for 10 min to remove nuclei and the other at 15,000 × *g* for 30 min to pellet other insoluble material. HLA complexes were captured by using 1 ml W6/32-conjugated immunoresin (5 mg/ml) prepared in a column format at a flow rate of 1.5 ml/min and washed by using subsequent runs with 50 mM Tris buffer (pH 8.0) containing first 150 mM NaCl, then 400 mM NaCl, and next no salt. HLA-peptide complexes were eluted by using 5 ml 10% acetic acid and dried.

### High-performance liquid chromatography fractionation.

Affinity column-eluted material was dried and resuspended in 120 μl buffer A (0.1% formic acid in water). Samples were loaded onto a 4.6- by 50-mm ProSwift RP-1S column (Thermo Scientific) and eluted by using a 500-μl/min flow rate over 10 min from 2% to 35% buffer B (0.1% formic acid in acetonitrile) in buffer A (0.1% formic acid in water), using an Ultimate 3000 high-performance liquid chromatography (HPLC) system (Thermo Scientific). One-milliliter fractions were collected from 2 to 15 min. Protein detection was performed at a 280-nm absorbance. Fractions that did not contain β_2_-microglobulin were combined, dried, and further analyzed by LC-MS/MS.

### Preparation of tryptic digests for proteomic analysis.

Cell pellets were lysed in lysis buffer (0.5% IGEPAL 630, 150 mM NaCl, 50 mM Tris [pH 8.0]) for 1 h with rotation at 4°C. Nuclei were removed by centrifugation for 10 min at 300 × *g*, and lysates were cleared by further centrifugation at 15,000 × *g* for 30 min. Total protein content was measured by using the Pierce BCA protein assay kit (Thermo Scientific) according to the manufacturer's instructions. Lysates were adjusted to the lowest protein concentration in lysis buffer, and an equivalent of 20 μg of total protein was loaded in 1× Laemmli buffer (50 mM Tris-HCl [pH 6.8], 10% glycerol, 2% SDS, 2.5% β-mercaptoethanol, 0.005% bromphenol blue) on a NuPAGE Novex 4 to 12% Bis-Tris protein gel (Invitrogen). Proteins were separated for 1 h at 200 V and visualized in the resulting gel by staining with InstantBlue (Expedeon). Complete gel lanes were excised and processed separately into three to four distinct samples. Gel slices were cut into 1-mm^2^ pieces and washed in 50% methanol–5% acetic acid in water to remove the InstantBlue stain. Gel pieces were dehydrated with acetonitrile and washed in 10 mM dithiothreitol–100 mM ammonium bicarbonate. Following a 30-min incubation, reduced cysteine residues were alkylated by the addition of 40 mM chloroacetamide in 100 mM ammonium bicarbonate for 30 min at room temperature. Trypsin digestion was carried out by the addition of 1 μg trypsin in 50 mM ammonium bicarbonate at 37°C overnight. Peptides were recovered from the gel pieces by extraction using 50% acetonitrile–5% acetic acid in water. Peptides were dried and suspended in 2% acetonitrile–0.1% formic acid in water for LC-MS/MS analysis.

### LC-MS/MS analysis.

Samples were suspended in 20 μl buffer A and analyzed on an Orbitrap Velos (Thermo Scientific) online instrument coupled to an Acquity Nano ultra-performance liquid chromatography (UPLC) (Waters) or on a TripleTOF 5600 (AB Sciex) instrument coupled to an Eksigent Ekspert nanoLC 400 cHiPLC system. For the Orbitrap Velos instrument, peptides were separated on a 25-cm BEH130 C_18_ column with a 1.7-mm particle size, using a linear gradient from 8% to 35% buffer B in buffer A at a flow rate of 250 nl/min (∼40 MPa) for 60 min. Peptides were introduced to the LTQ Orbitrap Velos mass spectrometer (Thermo Scientific) using a nano-electrospray ionization (nanoESI) source. Subsequent isolation and collision-induced dissociation (CID) were performed on the 20 most abundant ions per full MS scan using an isolation width of 1.5 Da. All fragmented precursor ions were actively excluded from repeated selection for 15 s. For the TripleTOF 5600 instrument, peptides were separated on a 15-cm by 75-μm ChromXP C_18_-CL column (Eksigent) with a linear gradient from 8% to 35% buffer B in buffer A at a flow rate of 300 nl/min (∼13 MPa) for 60 min. Peptides were introduced to the mass spectrometer by using a nanoESI source. Subsequent isolation and ramped-energy CID were performed on the 30 most abundant ions per full MS scan using a unit isolation width of ∼0.7 Da. All fragmented precursor ions were actively excluded from repeated selection for 15 s.

### MS data analysis interpretation.

Sequence interpretations of MS/MS spectra were performed by using a database containing all annotated human Swiss-Prot entries and MVA entries from NCBI combined with the sequence for the HIVconsv immunogen (30,581 sequences; 17,080,496 residues [February 2013]) by using PEAKS 7 (Bioinformatics Solutions Inc.) (32) at a false discovery rate (FDR) threshold of <5% with the following parameters: no enzyme specificity, with SILAC K4 or K8 and R10 as variable modifications for the SILAC experiments or otherwise no modifications; MS peptide tolerance of 5 ppm and MS/MS tolerance of 0.5 Da for the Orbitrap Velos instrument and MS peptide tolerance of 30 ppm and MS/MS tolerance of 0.05 Da for the TripleTOF instrument. Quantitation of all peptide signals was performed with Progenesis QI (Nonlinear Dynamics) or PEAKS 7. The correlation between HIVconsv peptide and protein abundances was evaluated by using Pearson correlation with GraphPad Prism 6.03 software. For individual peptide correlation factor calculations, peptide and protein abundances as determined by using Progenesis QI were first averaged across two technical replicate LC-MS/MS runs. For each protein, HLA-associated peptide abundances obtained from differently charged precursors were averaged for each time point, and Spearman's rank correlation coefficient with the correlating protein abundance in the cell lysate during the time course was computed across the four time points (0, 1.5, 2.5, and 3.5 h postinfection [hpi]).

### IFN-γ ELISPOT assay and peptide mapping.

Ethical and regulatory approvals to conduct the HIV-CORE 002 trial were obtained from the National Research Ethics Service Committee West London (10/H0707/52) and the UK Medicines and Healthcare Products Regulatory Agency (21584/0271/001). In order to map the responses of individual participants, short-term cell lines were derived from frozen peripheral blood mononuclear cell (PBMC) samples taken at week 28. The PBMCs were thawed, and an equal volume of RPMI supplemented with 10% FCS (RPMI10) plus 50 U/ml Benzonase nuclease (Novagen) warmed to 37°C was added dropwise. Cells were expanded by using pools 1 to 6 of HIVconsv-derived 15-mer peptides overlapping by 11 amino acids (aa) (22) for 10 days, rested, and tested by a gamma interferon (IFN-γ) enzyme-linked immunosorbent spot (ELISPOT) assay as described previously (27). Thus, ELISPOT plates (catalog number S5EJ044I10; Merck Millipore) prewetted for 1 min with 15 μl of 35% ethanol were coated overnight at +4°C with anti-IFN-γ antibody (10 μg/ml in PBS) (clone 1-D1K; Mabtech). Prior to use, plates were washed with PBS and blocked with RPMI10 for a minimum of 1 h at 37°C. The PBMCs were plated out at 4 × 10^4^ cells/well in 50 μl of RPMI10. Individual peptide responses were detected in duplicate wells with a peptide concentration of 2 μg/ml. Negative (no-peptide) and positive-control wells contained cells cultured in RPMI10 supplemented with 0.45% dimethyl sulfoxide (DMSO) and 10 μg/ml phytohemagglutinin (PHA) (Sigma-Aldrich), respectively. The cells were incubated overnight at 37°C in 5% CO_2_. Spots were visualized by using biotin anti-IFN-γ combined with streptavidin-alkaline phosphate (both from Mabtech), and the color was developed by using the substrate 5-bromo-4-chloro-3-indolyl-phosphate *p*-nitro blue tetrazolium chloride (BCIP/NBT^Plus^; Mabtech). The reaction was stopped after 5 min by washing under a tap. The plates were air dried overnight, and the spots were counted by using an AID ELISpot reader and version 5.0 software (AID GmbH). The average number of spot-forming units (SFU) in no-peptide wells was subtracted from the numbers in test wells, and the results were expressed as the median net SFU/10^6^ PBMCs.

## RESULTS

### Kinetics of HIVconsv protein expression in MVA.HIVconsv-infected cells.

**FIG 1:**
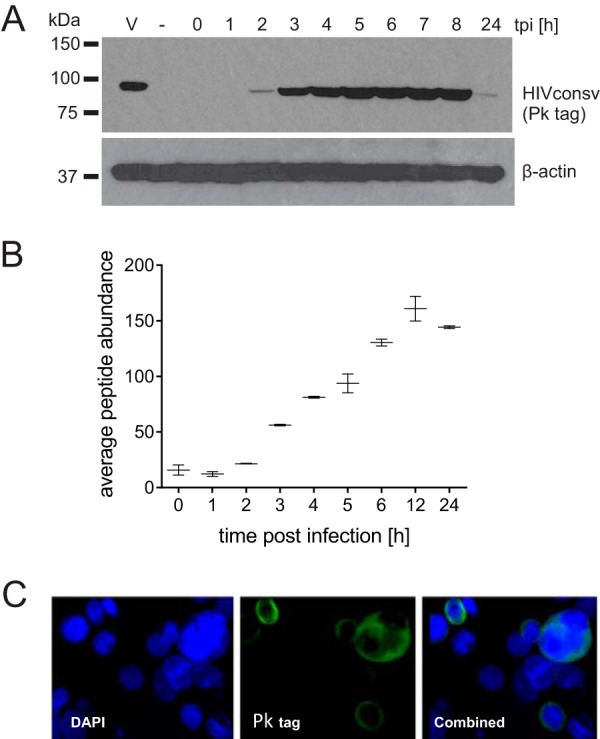
HIVconsv protein expression dynamics using MVA as a delivery vector. HIVconsv expression was monitored in cells infected with MVA.HIVconsv for the indicated durations by Western blotting using a V5 antibody (A), label-free quantitative LC-MS/MS (average abundance of 4 tryptic peptides) (B), and immunofluorescence at 24 hpi (C).

The HIVconsv gene is inserted into the thymidine kinase locus of the MVA genome under the control of the early/late 7.5 promoter. Jurkat cells were chosen as a model cell line for analysis of the HLA-associated peptidome, as they are easily expandable in cell culture, which includes the possibility of transformation with labeled amino acids, and they exhibit good levels of HLA class I expression. Before analysis of the immunopeptidome of MVA.HIVconsv-infected Jurkat cells, the levels of the whole-protein HIVconsv expression was assessed in a time course employing a C-terminal Pk tag recognized by monoclonal antibody (MAb) (33) in a Western blot analysis. This indicated that the HIVconsv protein reached a peak level by 5 h, maintained this level for at least 8 h, and almost disappeared by 24 hpi (Fig. 1A). To quantify the expression of the HIVconsv protein more accurately, protein levels were monitored following MVA.HIVconsv infection of Jurkat cells by using Q-MS analyses of trypsin-digested whole-cell protein extracts. The amounts of HIVconsv protein peaked at 12 hpi (Fig. 1B). Both the Western blot and quantitative LC-MS/MS techniques yielded similar results, and notably, both methods detected the HIVconsv protein with comparable sensitivities. The cytoplasmic localization of the HIVconsv protein was confirmed by immunofluorescence at 24 hpi (Fig. 1C). The half-life of the HIVconsv protein was previously determined by pulse-chase experiments using DNA expression vectors and Semliki Forest virus replicons to be 1 to 2 h (21; our unpublished observations). It is notable that the HIVconsv protein was detected at maintained levels at 24 hpi by LC-MS/MS, whereas the signal faded substantially in the Western blot analysis. This may be due to a loss of the C-terminal Pk tag due to processing, which is essential for detection by immunoblotting but not for LC-MS/MS. Since the HIVconsv protein could be identified as early as 2 hpi, and it has been reported that MVA infection interferes with MHC peptide presentation and induces apoptosis (34), we concluded that the first 3.5 h after MVA.HIVconsv infection would be the most suitable window for studying the early kinetics of the HIVconsv-derived immunopeptidome.

### HIVconsv-derived immunopeptides were detected at 1.5 h postinfection.

**FIG 2:**
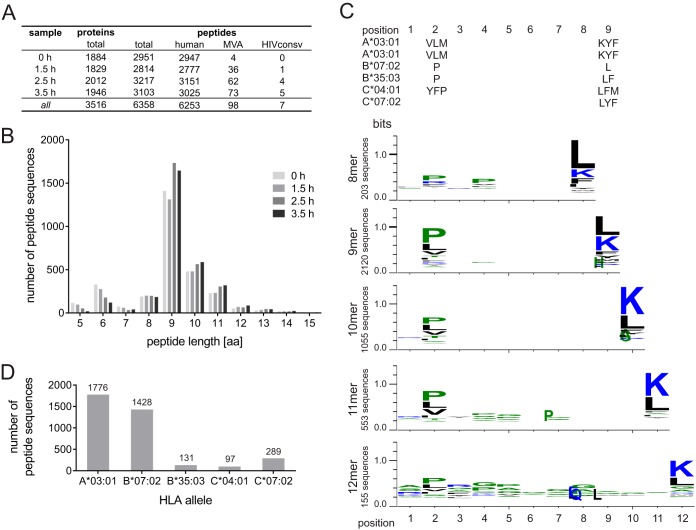
Characteristics of peptides eluted from Jurkat cells infected with MVA.HIVconsv. (A and B) Numbers of unique identified peptide sequences in the time course experiment (A) and their size distribution for each experiment (B). (C) Amino acid distribution of all eluted 8- to 12-mer peptides from all four samples in comparison to the known major anchor residues for the HLA allele of Jurkat cells, as listed above the graphs. The represented single-letter abbreviation for each amino acid is scaled by size according to the relative frequency of the amino acid at the indicated position in the eluted peptide (WebLogo 3.4). (D) Frequency of predicted binding of the identified peptide sequences to the HLA alleles of Jurkat cells, as determined with the NetMHC 3.4 server.

Next, we studied the kinetics of presentation of HLA class I-associated, HIVconsv-derived peptides. A total of 10^9^ Jurkat cells were infected with MVA.HIVconsv at an MOI of 10 for 0, 1.5, 2.5, and 3.5 h; peptide-loaded HLA class I complexes were immunopurified from cell lysates by using MAb W6/32; and the eluted peptides were purified via HPLC and analyzed by LC-MS/MS. The HIVconsv-derived peptide YKRWIILGLNK was detected as early as 1.5 h postinfection (Fig. 2A and Table 1). An average of 3,021 unique peptide sequences were identified for each time point, totaling 6,358 unique peptides. Of these peptides, 98 (see Table S1 in the supplemental material) were derived from the MVA vector, and 7 originated from the HIVconsv immunogen (Table 1). The other 6,253 identified sequences were derived from the human proteome. The HLA class I-eluted peptides ranged between 5 and 30 aa in length, and 5,080 of them (81%) were 8 to 12 aa long (Fig. 2B). Frequencies of individual amino acids in every epitope position were analyzed for all 8- and 12-mer peptides, and the HLA anchor amino acid preferences concurred with the HLA subtype of Jurkat cells (A*03:01, A*03:01, B*07:02, B*35:03, C*04:01, and C*07:02) (Fig. 2C) (35). Binding prediction with the NetMHC 3.4 server (36–38) identified 3,721 out of 5,080 peptides with a length of between 8 and 12 aa as binders to one of the six major alleles in Jurkat cells (Fig. 2D).

**TABLE 1:** HLA class I-associated peptides derived from the HIVconsv immunogen^*b*^

Sequence	Length (aa)	Position(s) in HXB2 (aa)	Positions in HIVconsv (aa)	Presence of exact match in LANL-HSD	NetMHC binding affinity (nM)	HLA allele	PEAKS score (−10logP)	Sample collection time(s) (h)
YKRWIILGLNK	11	Gag 262–272	58–68	No	11,608	A*03:01	15.30	1.5
KRWIILGLNK	10	Gag 263–272	59–68	Yes	6,379	A*03:01	16.35	2.5
IYKRWIILGLNK	12	Gag 261–272	57–68	No	1,472	A*03:01	15.32	3.5
IILGLNK	7	Gag 266–272	62–68	No	NA	A*03:01	14.82	2.5, 3.5
FPISPIETVPVKL	13	Pol 155–167	194–206	No	163 (WB)	B*07:02	56.52	3.5, 6
SPIETVPVKL	10	Pol 158–167	197–206	Yes	191 (WB)	B*07:02	41.33	2.5, 3.5, 6
AIFQSSMTK	9	Pol 313–321	351–360	Yes	20 (SB)	A*03:01	47.95	2.5, 3.5, 6
RKGGIGGYSAG	11	Pol 902–912	663–673	Yes	20,750	B*07:02	14.82	6
RTWKSLVK	8	Vif 19–26	412–419	No	39 (SB)	A*03:01	28.89	6
KIWPS-RWKPK^*a*^	10	Gag/Pol	131–140	NA	71 (WB)	A*03:01	19.25	6
KLTP-WVPAHK^*a*^	10	Env/Pol	518–527	NA	19 (SB)	A*03:01	40.52	6

a Peptides spanning a junction (-) between two conserved regions in the HIVconsv immunogen, thus creating a novel epitope not present on HIV-1-infected cells.

b LANL-HSD, Los Alamos National Laboratory HIV Sequence Database; SB, strong binder; WB, weak binder; NA, not applicable; −10logP, negative decadic logarithm of *P*, the probability that the identification is a random event.

### HIVconsv-derived peptide abundance correlates with levels of HIVconsv protein in the cell.

**FIG 3:**
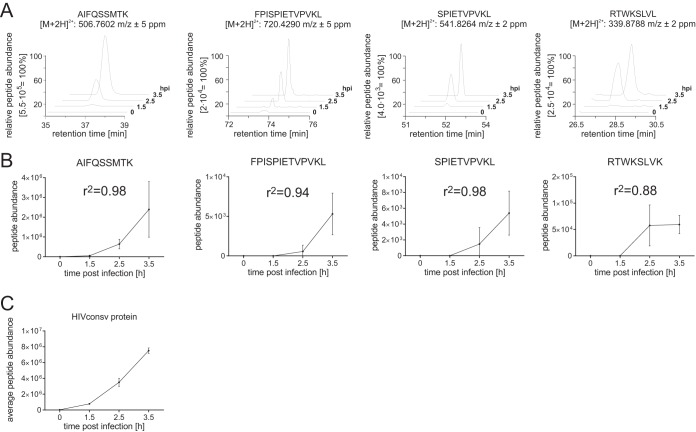
Correlation analysis of HIVconsv epitope and HIVconsv protein abundances. (A) Representative extracted ion chromatograms of the indicated peptide sequences plotted according to their retention time. (B) Average calculated abundances from duplicate analyses as determined by PEAKS. (C) *R*^2^ values calculated for each immunopeptide by using Pearson's correlation to total HIVconsv protein levels (average abundance of 63 tryptic peptides).

Four of the seven identified HIVconsv epitopes yielded MS signals that were of sufficient quality to quantify the peptide amounts at multiple times during the time course by using Q-MS. For all four peptides analyzed, the spectrum signals increased over time from 0 to 3.5 h (Fig. 3A and B), while confident (*P* ≤ 0.05; FDR ≤ 5%) identification of the peptide sequence was achieved at the 2.5- and 3.5-h time points. To assess the correlation between the abundances of the immunopeptides and their source proteins, whole HIVconsv protein levels at the four time points were determined by using a standard tryptic digest of the same infected-cell samples, followed by Q-MS analysis. It was found that the increase in HIVconsv-derived epitope presentation over time correlated with the increase of HIVconsv protein expression in the cytoplasm (Fig. 3B and C).

### Human vaccine recipients responded to the peptides identified by MS.

**FIG 4:**
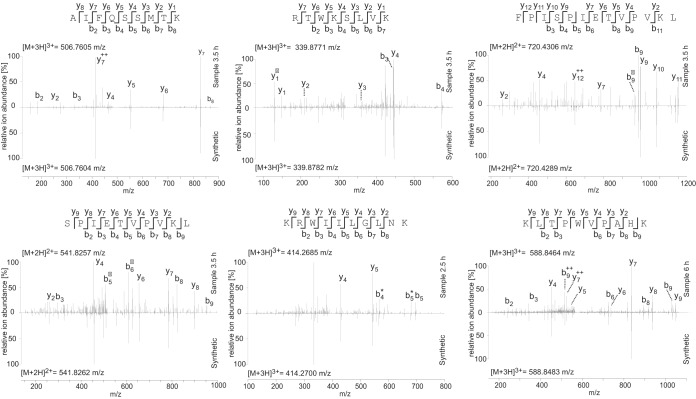
Spectral matches for HIVconsv-derived peptides. MS fragment spectra show the experimentally acquired spectrum of the indicated peptide in the indicated sample in comparison to a spectrum of the synthetic peptide counterpart acquired under the same conditions. Fragment ions are indicated for the peptide sequence above each spectrum, and the most intense ion mass peaks are labeled in the experimental spectrum as follows: b, singly charged N-terminal fragment ion; y, singly charged C-terminal fragment ion; 0, loss of H_2_O; *, loss of NH_2_; ++, doubly charged fragment ion. The detected mass of the intact peptide parent ion is stated for each spectrum.

**FIG 5:**
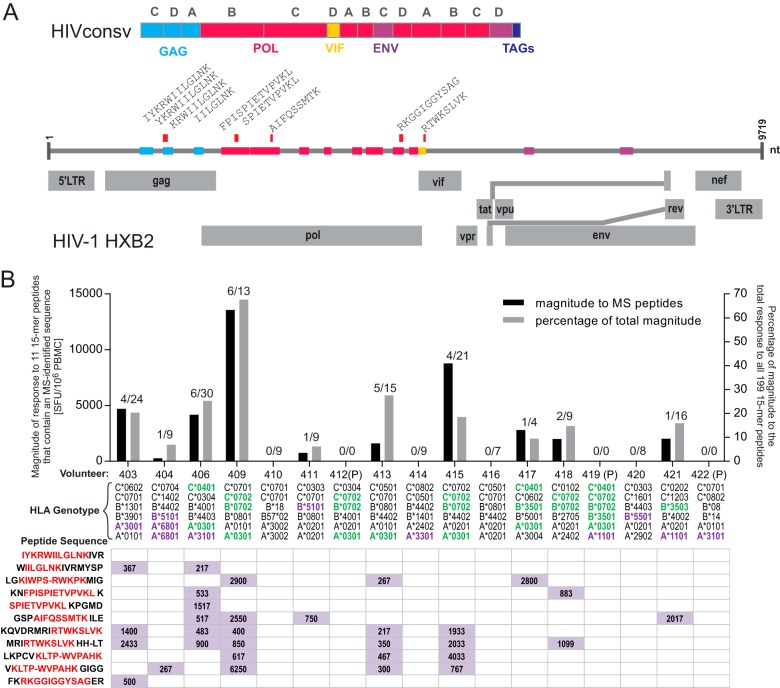
Responses in humans vaccinated with HIVconsv recognizing eluted, MS-identified peptides. (A) Schematic view of the HIVconsv immunogen, with the 14 conserved regions of HIV-1 that were combined in the immunogen represented as colored boxes and with the original HIV-1 region stated (Gag, Pol, Vif, and Env). Letters above the boxes (A, B, C, and D) indicate the clade of origin. The positions of eluted peptides identified in the HIVconsv immunogen are indicated by red bars. Genomic regions of the HXB2 strain are shown as gray rectangles. TAGs, epitope tag sequences; LTR, long terminal repeat; nt, nucleotides. (B) The magnitude of the response (in SFU/10^6^ PMBCs) is plotted for each individual, summed for all 15-mer peptides containing MS-identified sequences (left *y* axis), in addition to the percent magnitude compared to the total magnitude of responses to all 15-mer peptides spanning the full HIVconsv immunogen (right *y* axis). The breadth of the response is indicated by the number of peptides generating a response in each individual subject (MS-identified/total number of peptides, indicated above each bar). Individual responses to the indicated peptide sequences are shown below each graph column, and HLA genotypes for each volunteer are indicated. Matching HLA types are highlighted in green, and matching alleles from an identical HLA supertype are highlighted in violet. P, placebo recipient.

To increase the discovery of HIVconsv-derived epitopes, HLA class I-associated peptides were eluted from Jurkat cells 6 h after MVA.HIVconsv infection in two separate experiments. This longer infection led to the identification of four additional sequences, totaling 11 HIVconsv-derived peptides (Table 1 and Fig. 4 and 5A). In order to validate the identified HIVconsv-derived peptide sequences, the corresponding synthetic peptide standards were analyzed under identical conditions, and the obtained fragment spectra were compared. Spectral comparisons for 6 out of the 11 peptides identified are depicted in Fig. 4.

In the HIV-CORE 002 trial, the conserved immunogen HIVconsv was administered to healthy, HIV-1/2-uninfected volunteers in the United Kingdom, whereby all vaccine recipients developed T-cell responses to 15-mer peptides overlapping by 11 amino acids detected by an IFN-γ ELISPOT assay (27). In the two trial arms in which mapping to individual 15-mer peptides was carried out, 7 and 5 out of the 14 vaccine recipients shared at least 1 HLA allele or superallele, respectively (39), with the Jurkat cells (Fig. 5B). A total of 10 vaccine recipients responded to 15-mer peptides containing an immunopeptide eluted from MVA.HIVconsv-infected Jurkat cells (Fig. 5B). Every individual who shared at least one HLA allele with the cell line and 3 out of 5 individuals who shared at least one superallele responded to at least one 15-mer peptide containing an MS-identified immunopeptide. No placebo recipient responded to any HIVconsv-derived peptide (27). Overall, the magnitude of the response against 15-mer peptides that contained MS-identified peptide sequences was 15.2% of the total magnitude of the response against all 199 15-mer peptides, excluding the placebo recipients.

### MVA-derived epitopes can come from incoming virions rather than *de novo* synthesis.

The observed early presentation of MVA and HIVconsv immunopeptides can be due to the presentation of epitopes from incoming virion proteins directly or originate from newly synthesized gene products. To distinguish between these two sources of immunopeptides, we used stable isotope labeling of amino acids in cell culture (SILAC) to label all proteins with heavy lysine and arginine prior to infection of the cells with unlabeled MVA.HIVconsv. We then analyzed the immunopeptidome early, at 1.5 hpi, to maximize the probability of detecting HLA-associated peptides from incoming proteins. We identified two labeled immunopeptides originating from the MVA virus, IIGPMFSGK^8^ (+8.01 Da) from the thymidine kinase and MWGGGSSSGVK^8^ (+8.01 Da) from the hypothetical 5.6-kDa protein, which originated from newly synthesized protein (Table 2). Three unlabeled immunopeptides, RIISYNPPPK from the iActA-like 8-kDa cytoplasmic protein (iActA is a *Listeria ivanovii* protein involved in actin tail formation) and SPRIGDQL and ARPINGISY from the major core protein P4b, were detected and therefore likely originated from incoming protein material (Table 2). Four of the five immunopeptides, ARPINGISY and RIISYNPPPK at 1.5 hpi and IIGPMFSGK and SPRIGDQL at 2.5 hpi, had also been detected in the previous immunopeptidome experiment (see Table S1 in the supplemental material).

**TABLE 2:** MVA-derived epitope sequences at 1.5 hpi^*a*^

Sequence	Length (aa)	PEAKS score (−10logP)	Label mass (Da)	NetMHC binding affinity (nM)	HLA allele	GenBank protein accession no.	MVA protein
IIGPMFSGK^8^	9	31.93	+8.01	46 (SB)	A*03:01	CAM58264.1	Thymidine kinase
MWGGGSSSGVK^8^	11	40.23	+8.01	3,541	C*04:01	CAM58186.1	Hypothetical 5.6-kDa protein
RIISYNPPPK	10	29.40		30 (SB)	A*03:01	CAM58216.1	iActA-like 8-kDa cytoplasmic protein
SPRIGDQL	8	29.23		146 (WB)	B*07:02	CAM58292.1	Major core protein P4b
ARPINGISY	9	28.60		7,395	C*07:02	CAM58292.1	Major core protein P4b

a K^8^, heavy lysine, +8.01 Da; K, unlabeled lysine; R, unlabeled arginine; SB, strong binder; WB, weak binder; −10logP, negative decadic logarithm of *P*, the probability that the identification is a random event.

**FIG 6:**
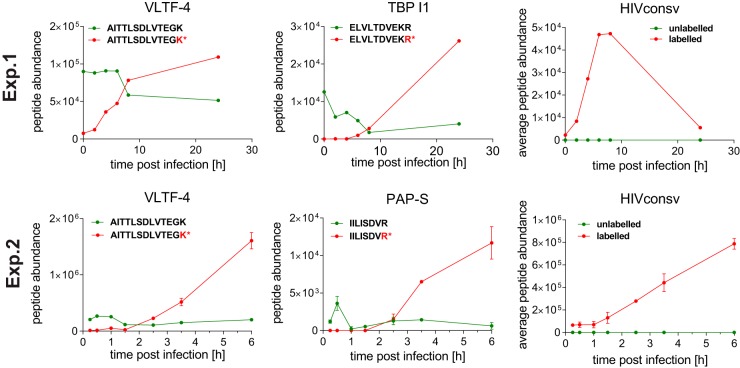
Determination of the origin of MVA.HIVconsv-specific protein using SILAC. Shown are the results from two independent experiments (top row, experiment 1 [Exp.1]; bottom row, experiment 2 [Exp.2]). Labeled peptide (newly made) abundances are plotted as red curves, and unlabeled (incoming virus particle-derived) peptide abundances are depicted in green. Single tryptic peptide abundances are plotted for the MVA proteins VLTF-4, TBP-I1, and PAP-S, as measured in both experiments. For the HIVconsv protein, average abundances of 5 (experiment 1) or 23 (experiment 2) tryptic peptides are plotted, reflecting the abundance of the HIVconsv protein at the indicated time points after infection with MVA.HIVconsv.

In order to monitor the amount of incoming virion proteins, we performed a time course of infection using SILAC-labeled Jurkat cells and unlabeled virus. We first analyzed samples at the 0-, 2-, 4-, 6-, 8-, 10-, and 24-hpi time points, and in a second experiment, we focused in greater detail on early-infection sampling at 0.25, 0.5, 1, 1.5, 2.5, 3.5, and 6 hpi. Protein material was isolated, digested with trypsin, and analyzed by MS. Signals of labeled and unlabeled peptides were quantified separately by using Progenesis QI, and abundances were monitored throughout the analyzed time course. One peptide each from three MVA-derived proteins, viral late transcription factor 4 (VLTF-4), the Cap-specific mRNA (nucleoside-2′-*O*-)-methyltransferase (PAP-S), and telomere-binding protein I1 (TBP-I1), was detected in both labeled and unlabeled forms (Fig. 6), indicating that a proportion of this material originated from incoming MVA virions. However, despite the detection of the HIVconsv protein in the purified MVA.HIVconsv stock (Fig. 1, lane V), HIVconsv-derived tryptic peptides were detected only in the labeled form, indicating *de novo* synthesis (Fig. 6). Thus, all the MS-detectable HIVconsv-derived immunopeptides most likely originated from newly synthesized HIVconsv protein in vaccine-infected cells.

### The immunopeptidome reflects changes of the cellular proteome upon MVA.HIVconsv infection.

**FIG 7:**
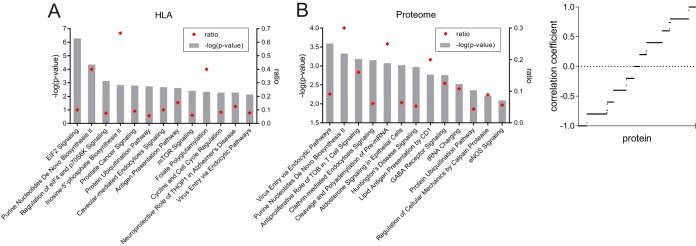
The immunopeptidome reflects changes of the cellular proteome in MVA.HIVconsv-infected cells. (A) The most relevant pathways that are affected by MVA.HIVconsv infection at 3.5 hpi, as identified by Ingenuity Pathway Analysis, for all proteins according to the abundances of the corresponding HLA-associated peptides (left) and the intracellular proteins (right), as determined by label-free quantitative LC-MS/MS. All changes in abundance were determined between the 3.5-h time point and the negative control. (B) Correlation factors between the trend of protein abundance and the trend of the HLA-associated peptide abundance at 0, 1.5, 2.5, and 3.5 hpi. A proportional correlation was assigned a value of +1, and an inverse correlation was assigned a value of −1. Factors are plotted for all proteins along the *x* axis, for which both protein and immunopeptide abundances were determined.

Infection of cells with viruses including MVA (40) results in a dramatic alteration of host cell protein expression. To assess the changes in the Jurkat cell proteome following MVA.HIVconsv infection and the resulting altered self-peptidome associated with the HLA class I molecules, proteomes and immunopeptidomes of uninfected and MVA.HIVconsv-infected cells were analyzed and compared by using Ingenuity Pathway Analysis (IPA) software (Fig. 7A). Generally, many similarities between protein and HLA peptide abundances were observed. The “virus entry via endocytic pathways,” “purine nucleotides *de novo* biosynthesis,” and “protein ubiquitination pathway” were among the most significantly affected pathways, reflected by both the cellular proteome and immunopeptidome. However, when correlating the protein abundance trend with the trend of the corresponding epitope presentation abundance throughout the analyzed time course, a range of proportional (+1 correlation factor) to antiproportional (−1 correlation factor) correlations and no correlation (0 correlation factor) were observed (Fig. 7B; see also Table S2 in the supplemental material). Interestingly, for any one protein for which multiple peptides were presented, correlation trends were not always consistent (see Table S2 in the supplemental material). These findings indicate that for each HLA-associated peptide, a specific generation-and-presentation pathway applies, and a general correlation between presentation and protein abundance is not feasible.

## DISCUSSION

Characterization and quantitation of pathogen-derived, HLA-restricted peptides over multiple time points are now feasible by using Q-MS (11). Here, we report a number of interesting observations from analyses of the HLA class I-associated immunopeptidome in human cells infected with the experimental vaccine MVA.HIVconsv, which aims to induce T-cell responses focused on conserved subdominant HIV-1 epitopes. First, peptides derived from conserved HIV-1 regions can be readily detected in association with HLA class I molecules. The earliest time that a conserved peptide was detected was 1.5 h after MVA.HIVconsv cell entry, and in 6 h, a total of 11 peptides were identified from the 806-aa-long HIVconsv protein. Four of the 11 conserved immunopeptides could be monitored over several time points, and their abundance in HLA class I associations reflected the expression levels of the whole HIVconsv protein in the cell. Although proteins delivered by the incoming recombinant MVA virions were detected, the detectable level of early HIVconsv protein was exclusively synthesized *de novo*. Finally, MVA.HIVconsv infection altered the composition of HLA class I-associated self-peptides, which corresponded only partially with host protein analysis using MS of a standard protein trypsin digest.

Cytotoxic T cells substantially contribute to the control of HIV-1 infection. There is a growing belief that effective T-cell responses might provide a key contribution to the control of HIV-1 infection, and (re)focusing T-cell responses on the conserved epitopes of the HIV-1 proteome might gain the necessary competitive advantage over HIV-1, particularly for therapeutic vaccination (13, 15, 41–43). Here, we demonstrate directly by LC-MS/MS analysis of the HLA class I-associated peptidome that conserved epitopes, which are subdominant during natural HIV-1 infection and therefore outcompeted by dominant, less protective determinants, can be readily detected on HLA complexes when taken out of the context of HIV-1 proteins and expressed from an artificial chimeric vaccine immunogen. Given the restricting HLAs, there was a good match between the MS-detected immunopeptides and responses induced by the same HIVconsv vaccine(s) in human volunteers (27). Furthermore, the epitope RKGGIGGYSAG was previously reported to be a target of T cells in a patient with high-level exposure who remained uninfected for >2 years (44). Also, the epitope IYKRWIILGLNK is conserved throughout the clades and has been identified as a cross-clade T-cell target in infected individuals (45). Thus, our direct characterization of peptides from MVA.HIVconsv-infected cells supports the conserved-region strategy for the development of T-cell vaccines (21, 22, 30, 33).

MVA has a complex genome carrying 193 open reading frames (46) thought to code for functional proteins. These open reading frames are classified as early, intermediate, and late based on the timing of their expression, and this is in turn determined by the promoters driving the transcription of the corresponding genes. Multiple mechanisms interfere with the presentation of late T-cell epitopes (47–52). Here, we report the identification of 98 MVA-derived peptides eluted from HLA class I. In most mammalian cells, the growth of MVA is blocked late in the replication cycle; nevertheless, pathogen-derived transgenes, particularly those which are expressed early, can very strongly boost both existing antibody and CD8^+^ T-cell responses. The magnitude of induced transgene-specific responses can be dictated by the insertion locus in the MVA genome and the strength of the promoter used to drive the transcription of the transgene product (53–55). Promoter P7.5, employed here to express HIVconsv, was the weakest of the modified H5 and synthetic promoters compared (56).

We monitored changes in the abundances of presented peptides during the first hours following MVA.HIVconsv infection using mass spectrometry. Despite detectable HIVconsv protein levels in the MVA.HIVconsv stock, our data demonstrate that all HIVconsv protein detected in infected cells was synthesized *de novo*. Since protein levels correlated with the abundance trends for the detected HIVconsv-derived immunopeptides early in infection, peptide presentation likely originated from these *de novo*-synthesized protein precursors. We identified two immunopeptides of MVA proteins that were derived from neosynthesized precursors and were therefore likely generated from either defective ribosomal products (DRiPs), which are generated during translation (57); rapid turnover of full-length protein following neosynthesis; or both. A similar dependence on neosynthesis was shown previously for the presentation of an immunodominant epitope of the highly stable lymphocytic choriomeningitis virus nucleoprotein, which was no longer presented when translation was abrogated (58). A direct interrogation of whether “retirees,” which are proteins turned over at the end of their life span, or DRiPs represented the major source for peptide presentation identified the latter as the major contributor to the presentation of viral antigens (59) and the self-MHC class I peptide repertoire (60–62). Furthermore, in mouse thymocytes, the MHC class I repertoire was molded by the transcriptome, as a clear enrichment of highly abundant mRNAs encoding MHC class I-associated peptides relative to low-abundance mRNAs was reported (63), which further supports the importance of neosynthesis for peptide presentation. More recently, it was suggested that MHC-associated peptides derive mainly from the “pioneer” round of translation, which takes place before the steady-state translation of mRNA is initiated and which is important for mRNA quality control (64). However, it has also been shown that full-length proteins can be a source for MHC class I peptide presentation (65, 66). In compliance with this, we detected 3 unlabeled MVA immunopeptides in heavy-amino-acid-labeled cells that were infected with unlabeled virus particles, strongly suggesting presentation of degraded protein material independent of neosynthesis. Therefore, our data further suggest that the correlation between protein abundance and presentation of the corresponding peptide is variable and needs to be interrogated separately for each epitope, as suggested previously (11).

We also confirmed that infection of cells with MVA altered the host cell proteome and the self-MHC class I peptide repertoire dramatically, supporting and further expanding previous observations for MVA infection (40). Similar changes in the self-MHC class I peptide repertoire have also been shown in the context of other viral infections, such as HIV-1 (67) and influenza A virus (8) infections, and other modulators of cellular metabolism, such as rapamycin (68).

In conclusion, the increased sensitivity of LC-MS/MS and better software packages for peptide identification continue to improve the direct identification of HLA class I-associated epitopes derived from important human pathogens. This has ramifications for the development of not only HIV-1 vaccines but also vaccines against other complex pathogens, which are still awaiting identification of relevant T-cell targets.

## Supplementary Material

Supplemental material

## References

[1] Shimonkevitz R, Colon S, Kappler JW, Marrack P, Grey HM 1984 Antigen recognition by H-2-restricted T cells. II. A tryptic ovalbumin peptide that substitutes for processed antigen. J Immunol 133:2067–2074. 6332146

[2] Townsend AR, Rothbard J, Gotch FM, Bahadur G, Wraith D, McMichael AJ 1986 The epitopes of influenza nucleoprotein recognized by cytotoxic T lymphocytes can be defined with short synthetic peptides. Cell 44:959–968. doi:10.1016/0092-8674(86)90019-X. 2420472

[3] Brehm MA, Pinto AK, Daniels KA, Schneck JP, Welsh RM, Selin LK 2002 T cell immunodominance and maintenance of memory regulated by unexpectedly cross-reactive pathogens. Nat Immunol 3:627–634. doi:10.1038/ni806. 12055626

[4] Yewdell JW 2006 Confronting complexity: real-world immunodominance in antiviral CD8+ T cell responses. Immunity 25:533–543. doi:10.1016/j.immuni.2006.09.005. 17046682

[5] Yewdell JW, Bennink JR 1999 Immunodominance in major histocompatibility complex class I-restricted T lymphocyte responses. Annu Rev Immunol 17:51–88. doi:10.1146/annurev.immunol.17.1.51. 10358753

[6] McMichael A, Klenerman P, Rowland-Jones S, Gotch F, Moss P 1995 Recognition of viral antigens at the cell surface. Cancer Surv 22:51–62. 7536629

[7] Johnson KL, Ovsyannikova IG, Mason CJ, Bergen HR III, Poland GA 2009 Discovery of naturally processed and HLA-presented class I peptides from vaccinia virus infection using mass spectrometry for vaccine development. Vaccine 28:38–47. doi:10.1016/j.vaccine.2009.09.126. 19822231 PMC2787804

[8] Wahl A, Schafer F, Bardet W, Hildebrand WH 2010 HLA class I molecules reflect an altered host proteome after influenza virus infection. Hum Immunol 71:14–22. doi:10.1016/j.humimm.2009.08.012. 19748539 PMC2795087

[9] Wolk B, Trautwein C, Buchele B, Kersting N, Blum HE, Rammensee HG, Cerny A, Stevanovic S, Moradpour D, Brass V 2012 Identification of naturally processed hepatitis C virus-derived major histocompatibility complex class I ligands. PLoS One 7:e29286. doi:10.1371/journal.pone.0029286. 22235280 PMC3250420

[10] Mommen GP, Frese CK, Meiring HD, van Gaans-van den Brink J, de Jong AP, van Els CA, Heck AJ 2014 Expanding the detectable HLA peptide repertoire using electron-transfer/higher-energy collision dissociation (EThcD). Proc Natl Acad Sci U S A 111:4507–4512. doi:10.1073/pnas.1321458111. 24616531 PMC3970485

[11] Croft NP, Smith SA, Wong YC, Tan CT, Dudek NL, Flesch IE, Lin LC, Tscharke DC, Purcell AW 2013 Kinetics of antigen expression and epitope presentation during virus infection. PLoS Pathog 9:e1003129. doi:10.1371/journal.ppat.1003129. 23382674 PMC3561264

[12] Walker B, McMichael A 2012 The T-cell response to HIV. Cold Spring Harb Perspect Med 2:a007054. doi:10.1101/cshperspect.a007054. 23002014 PMC3543107

[13] Hanke T 2014 Conserved immunogens in prime-boost strategies for the next-generation HIV-1 vaccines. Expert Opin Biol Ther 14:601–616. doi:10.1517/14712598.2014.885946. 24490585

[14] Walker BD, Burton DR 2008 Toward an AIDS vaccine. Science 320:760–764. doi:10.1126/science.1152622. 18467582

[15] Letourneau S, Im E-J, Mashishi T, Brereton C, Bridgeman A, Yang H, Dorrell L, Dong T, Korber B, McMichael AJ, Hanke T 2007 Design and pre-clinical evaluation of a universal HIV-1 vaccine. PLoS One 2:e984. doi:10.1371/journal.pone.0000984. 17912361 PMC1991584

[16] Altfeld M, Addo MM, Rosenberg ES, Hecht FM, Lee PK, Vogel M, Yu XG, Draenert R, Johnston MN, Strick D, Allen TM, Feeney ME, Kahn JO, Sekaly RP, Levy JA, Rockstroh JK, Goulder PJ, Walker BD 2003 Influence of HLA-B57 on clinical presentation and viral control during acute HIV-1 infection. AIDS 17:2581–2591. doi:10.1097/00002030-200312050-00005. 14685052

[17] Ferguson AL, Mann JK, Omarjee S, Ndung'u T, Walker BD, Chakraborty AK 2013 Translating HIV sequences into quantitative fitness landscapes predicts viral vulnerabilities for rational immunogen design. Immunity 38:606–617. doi:10.1016/j.immuni.2012.11.022. 23521886 PMC3728823

[18] Kelleher AD, Long C, Holmes EC, Allen RL, Wilson J, Conlon C, Workman C, Shaunak S, Olson K, Goulder P, Brander C, Ogg G, Sullivan JS, Dyer W, Jones I, McMichael AJ, Rowland-Jones S, Phillips RE 2001 Clustered mutations in HIV-1 gag are consistently required for escape from HLA-B27-restricted cytotoxic T lymphocyte responses. J Exp Med 193:375–386. doi:10.1084/jem.193.3.375. 11157057 PMC2195921

[19] Leslie AJ, Pfafferott KJ, Chetty P, Draenert R, Addo MM, Feeney M, Tang Y, Holmes EC, Allen T, Prado JG, Altfeld M, Brander C, Dixon C, Ramduth D, Jeena P, Thomas SA, John AS, Roach TA, Kupfer B, Luzzi G, Edwards A, Taylor G, Lyall H, Tudor-Williams G, Novelli V, Martinez-Picado J, Kiepiela P, Walker BD, Goulder PJ 2004 HIV evolution: CTL escape mutation and reversion after transmission. Nat Med 10:282–289. doi:10.1038/nm992. 14770175

[20] Im E-J, Hong JP, Roshorm Y, Bridgeman A, Létourneau S, Liljeström P, Potash MJ, Volsky DJ, McMichael AJ, Hanke T 2011 Protective efficacy of serially up-ranked subdominant CD8+ T cell epitopes against virus challenges. PLoS Pathog 7:e1002041. doi:10.1371/journal.ppat.1002041. 21625575 PMC3098219

[21] Knudsen ML, Mbewe-Mvula A, Rosario M, Johansson DX, Kakoulidou M, Bridgeman A, Reyes-Sandoval A, Nicosia A, Ljungberg K, Hanke T, Liljestrom P 2012 Superior induction of T cell responses to conserved HIV-1 regions by electroporated alphavirus replicon DNA compared to conventional plasmid DNA vaccine. J Virol 86:4082–4090. doi:10.1128/JVI.06535-11. 22318135 PMC3318663

[22] Ondondo B, Abdul-Jawad S, Bridgeman A, Hanke T 2014 Characterization of T-cell responses to conserved regions of the HIV-1 proteome in BALB/c mice. Clin Vaccine Immunol 21:1565–1572. doi:10.1128/CVI.00587-14. 25230940 PMC4248756

[23] Rosario M, Borthwick N, Stewart-Jones GB, Mbewe-Mwula A, Bridgeman A, Colloca S, Montefiori D, McMichael AJ, Nicosia A, Drijfhout JW, Melief CJM, Hanke T 2012 Prime-boost regimens with adjuvanted synthetic long peptides elicit T cells and antibodies to conserved regions of HIV-1 in macaques. AIDS 26:275–284. doi:10.1097/QAD.0b013e32834ed9b2. 22095198

[24] Rosario M, Bridgeman A, Quakkelaar ED, Quigley MF, Hill BJ, Knudsen ML, Ammendola V, Ljungberg K, Borthwick N, Im EJ, McMichael AJ, Drijfhout JW, Greenaway HY, Venturi V, Douek DC, Colloca S, Liljestrom P, Nicosia A, Price DA, Melief CJ, Hanke T 2010 Long peptides induce polyfunctional T cells against conserved regions of HIV-1 with superior breadth to single-gene vaccines in macaques. Eur J Immunol 40:1973–1984. doi:10.1002/eji.201040344. 20468055

[25] Im E-J, Hanke T 2004 MVA as a vector for vaccines against HIV-1. Expert Rev Vaccines 3:S89–S97. doi:10.1586/14760584.3.4.S89. 15285708

[26] Mayr A, Hochstein-Mintzel V, Stickl H 1975 Abstammung, Eigenschaften und Verwendung des attenuierten Vaccinia-Stammes MVA. Infection 105:6–14.

[27] Borthwick N, Ahmed T, Ondondo B, Hayes P, Rose A, Ebrahimsa U, Hayton EJ, Black A, Bridgeman A, Rosario M, Hill AV, Berrie E, Moyle S, Frahm N, Cox J, Colloca S, Nicosia A, Gilmour J, McMichael AJ, Dorrell L, Hanke T 2014 Vaccine-elicited human T cells recognizing conserved protein regions inhibit HIV-1. Mol Ther 22:464–475. doi:10.1038/mt.2013.248. 24166483 PMC3911893

[28] Dorrell L, Yang H, Ondondo B, Dong T, di Gleria K, Suttill A, Conlon C, Brown D, Williams P, Bowness P, Goonetilleke N, Rostron T, Rowland-Jones S, Hanke T, McMichael AJ 2006 Expansion and diversification of virus-specific T cells following immunization of human immunodeficiency virus type 1 (HIV-1)-infected individuals with a recombinant modified vaccinia virus Ankara/HIV-1 Gag vaccine. J Virol 80:4705–4716. doi:10.1128/JVI.80.10.4705-4716.2006. 16641264 PMC1472080

[29] Hanke T, Goonetilleke N, McMichael AJ, Dorrell L 2007 Clinical experience with plasmid DNA- and modified vaccinia vaccine Ankara (MVA)-vectored HIV-1 clade A vaccine inducing T cells. J Gen Virol 88:1–12. doi:10.1099/vir.0.82493-0. 17170430

[30] McShane H, Pathan AA, Sander CR, Keating SM, Gilbert SC, Huygen K, Fletcher HA, Hill AV 2004 Recombinant modified vaccinia virus Ankara expressing antigen 85A boosts BCG-primed and naturally acquired antimycobacterial immunity in humans. Nat Med 10:1240–1244. doi:10.1038/nm1128. 15502839

[31] Sheehy SH, Duncan CJ, Elias SC, Choudhary P, Biswas S, Halstead FD, Collins KA, Edwards NJ, Douglas AD, Anagnostou NA, Ewer KJ, Havelock T, Mahungu T, Bliss CM, Miura K, Poulton ID, Lillie PJ, Antrobus RD, Berrie E, Moyle S, Gantlett K, Colloca S, Cortese R, Long CA, Sinden RE, Gilbert SC, Lawrie AM, Doherty T, Faust SN, Nicosia A, Hill AV, Draper SJ 2012 ChAd63-MVA-vectored blood-stage malaria vaccines targeting MSP1 and AMA1: assessment of efficacy against mosquito bite challenge in humans. Mol Ther 20:2355–2368. doi:10.1038/mt.2012.223. 23089736 PMC3519995

[32] Zhang J, Xin L, Shan B, Chen W, Xie M, Yuen D, Zhang W, Zhang Z, Lajoie GA, Ma B 2012 PEAKS DB: de novo sequencing assisted database search for sensitive and accurate peptide identification. Mol Cell Proteomics 11:M111.010587. doi:10.1074/mcp.M111.010587. PMC3322562 22186715

[33] Hanke T, Botting C, Green EA, Szawlowski PW, Rud E, Randall RE 1994 Expression and purification of nonglycosylated SIV proteins, and their use in induction and detection of SIV-specific immune responses. AIDS Res Hum Retroviruses 10:665–674. doi:10.1089/aid.1994.10.665. 8074930

[34] Guzman E, Cubillos-Zapata C, Cottingham MG, Gilbert SC, Prentice H, Charleston B, Hope JC 2012 Modified vaccinia virus Ankara-based vaccine vectors induce apoptosis in dendritic cells draining from the skin via both the extrinsic and intrinsic caspase pathways, preventing efficient antigen presentation. J Virol 86:5452–5466. doi:10.1128/JVI.00264-12. 22419811 PMC3347273

[35] Crooks GE, Hon G, Chandonia JM, Brenner SE 2004 WebLogo: a sequence logo generator. Genome Res 14:1188–1190. doi:10.1101/gr.849004. 15173120 PMC419797

[36] Lundegaard C, Lamberth K, Harndahl M, Buus S, Lund O, Nielsen M 2008 NetMHC-3.0: accurate Web accessible predictions of human, mouse and monkey MHC class I affinities for peptides of length 8-11. Nucleic Acids Res 36:W509–W512. doi:10.1093/nar/gkn202. 18463140 PMC2447772

[37] Lundegaard C, Lund O, Nielsen M 2008 Accurate approximation method for prediction of class I MHC affinities for peptides of length 8, 10 and 11 using prediction tools trained on 9mers. Bioinformatics 24:1397–1398. doi:10.1093/bioinformatics/btn128. 18413329

[38] Nielsen M, Lundegaard C, Worning P, Lauemoller SL, Lamberth K, Buus S, Brunak S, Lund O 2003 Reliable prediction of T-cell epitopes using neural networks with novel sequence representations. Protein Sci 12:1007–1017. doi:10.1110/ps.0239403. 12717023 PMC2323871

[39] Sidney J, Grey HM, Kubo RT, Sette A 1996 Practical, biochemical and evolutionary implications of the discovery of HLA class I supermotifs. Immunol Today 17:261–266. doi:10.1016/0167-5699(96)80542-1. 8962628

[40] Guerra S, Gonzalez JM, Climent N, Reyburn H, Lopez-Fernandez LA, Najera JL, Gomez CE, Garcia F, Gatell JM, Gallart T, Esteban M 2010 Selective induction of host genes by MVA-B, a candidate vaccine against HIV/AIDS. J Virol 84:8141–8152. doi:10.1128/JVI.00749-10. 20534857 PMC2916545

[41] Altfeld M, Allen TM 2006 Hitting HIV where it hurts: an alternative approach to HIV vaccine design. Trends Immunol 27:504–510. doi:10.1016/j.it.2006.09.007. 16997629

[42] Kunwar P, Hawkins N, Dinges WL, Liu Y, Gabriel EE, Swan DA, Stevens CE, Maenza J, Collier AC, Mullins JI, Hertz T, Yu X, Horton H 2013 Superior control of HIV-1 replication by CD8+ T cells targeting conserved epitopes: implications for HIV vaccine design. PLoS One 8:e64405. doi:10.1371/journal.pone.0064405. 23741326 PMC3669284

[43] Rolland M, Nickle DC, Mullins JI 2007 HIV-1 group M conserved elements vaccine. PLoS Pathog 3:e157. doi:10.1371/journal.ppat.0030157. 18052528 PMC2098811

[44] Liu Y, Woodward A, Zhu H, Andrus T, McNevin J, Lee J, Mullins JI, Corey L, McElrath MJ, Zhu T 2009 Preinfection human immunodeficiency virus (HIV)-specific cytotoxic T lymphocytes failed to prevent HIV type 1 infection from strains genetically unrelated to viruses in long-term exposed partners. J Virol 83:10821–10829. doi:10.1128/JVI.00839-09. 19706711 PMC2753147

[45] Gudmundsdotter L, Bernasconi D, Hejdeman B, Sandstrom E, Alaeus A, Lidman K, Ensoli B, Wahren B, Butto S 2008 Cross-clade immune responses to Gag p24 in patients infected with different HIV-1 subtypes and correlation with HLA class I and II alleles. Vaccine 26:5182–5187. doi:10.1016/j.vaccine.2008.03.094. 18479789

[46] Antoine G, Scheiflinger F, Dorner F, Falkner FG 1998 The complete genomic sequence of the modified vaccinia Ankara strain: comparison with other orthopoxviruses. Virology 244:365–396. doi:10.1006/viro.1998.9123. 9601507

[47] Dasgupta A, Hammarlund E, Slifka MK, Fruh K 2007 Cowpox virus evades CTL recognition and inhibits the intracellular transport of MHC class I molecules. J Immunol 178:1654–1661. doi:10.4049/jimmunol.178.3.1654. 17237415

[48] Kastenmuller W, Gasteiger G, Gronau JH, Baier R, Ljapoci R, Busch DH, Drexler I 2007 Cross-competition of CD8+ T cells shapes the immunodominance hierarchy during boost vaccination. J Exp Med 204:2187–2198. doi:10.1084/jem.20070489. 17709425 PMC2118691

[49] Liu L, Chavan R, Feinberg M 2008 Dendritic cells are preferentially targeted among hematolymphocytes by modified vaccinia virus Ankara and play a key role in the induction of virus-specific T cell responses in vivo. BMC Immunol 9:15. doi:10.1186/1471-2172-9-15. 18412969 PMC2359732

[50] Rehm KE, Connor RF, Jones GJ, Yimbu K, Mannie MD, Roper RL 2009 Vaccinia virus decreases major histocompatibility complex (MHC) class II antigen presentation, T-cell priming, and peptide association with MHC class II. Immunology 128:381–392. doi:10.1111/j.1365-2567.2009.03120.x. 20067538 PMC2770686

[51] Townsend A, Bastin J, Gould K, Brownlee G, Andrew M, Coupar B, Boyle D, Chan S, Smith G 1988 Defective presentation to class I-restricted cytotoxic T lymphocytes in vaccinia-infected cells is overcome by enhanced degradation of antigen. J Exp Med 168:1211–1224. doi:10.1084/jem.168.4.1211. 2459295 PMC2189091

[52] Webb TJ, Litavecz RA, Khan MA, Du W, Gervay-Hague J, Renukaradhya GJ, Brutkiewicz RR 2006 Inhibition of CD1d1-mediated antigen presentation by the vaccinia virus B1R and H5R molecules. Eur J Immunol 36:2595–2600. doi:10.1002/eji.200636024. 16981180

[53] Chakrabarti S, Sisler JR, Moss B 1997 Compact, synthetic, vaccinia virus early/late promoter for protein expression. Biotechniques 23:1094–1097. 9421642 10.2144/97236st07

[54] Wang Z, Martinez J, Zhou W, La Rosa C, Srivastava T, Dasgupta A, Rawal R, Li Z, Britt WJ, Diamond D 2010 Modified H5 promoter improves stability of insert genes while maintaining immunogenicity during extended passage of genetically engineered MVA vaccines. Vaccine 28:1547–1557. doi:10.1016/j.vaccine.2009.11.056. 19969118 PMC2821965

[55] Wyatt LS, Shors ST, Murphy BR, Moss B 1996 Development of a replication-deficient recombinant vaccinia virus vaccine effective against parainfluenza virus 3 infection in an animal model. Vaccine 14:1451–1458. doi:10.1016/S0264-410X(96)00072-2. 8994321

[56] Hopkins R, Bridgeman A, Joseph J, Gilbert SC, McShane H, Hanke T 2011 Dual neonate vaccine platform against HIV-1 and M. tuberculosis. PLoS One 6:e20067. doi:10.1371/journal.pone.0020067. 21603645 PMC3094449

[57] Yewdell JW, Reits E, Neefjes J 2003 Making sense of mass destruction: quantitating MHC class I antigen presentation. Nat Rev Immunol 3:952–961. doi:10.1038/nri1250. 14647477

[58] Khan S, de Giuli R, Schmidtke G, Bruns M, Buchmeier M, van den Broek M, Groettrup M 2001 Cutting edge: neosynthesis is required for the presentation of a T cell epitope from a long-lived viral protein. J Immunol 167:4801–4804. doi:10.4049/jimmunol.167.9.4801. 11673482

[59] Cardinaud S, Starck SR, Chandra P, Shastri N 2010 The synthesis of truncated polypeptides for immune surveillance and viral evasion. PLoS One 5:e8692. doi:10.1371/journal.pone.0008692. 20098683 PMC2809100

[60] Dolan BP, Li L, Veltri CA, Ireland CM, Bennink JR, Yewdell JW 2011 Distinct pathways generate peptides from defective ribosomal products for CD8+ T cell immunosurveillance. J Immunol 186:2065–2072. doi:10.4049/jimmunol.1003096. 21228349 PMC3408966

[61] Dolan BP, Bennink JR, Yewdell JW 2011 Translating DRiPs: progress in understanding viral and cellular sources of MHC class I peptide ligands. Cell Mol Life Sci 68:1481–1489. doi:10.1007/s00018-011-0656-z. 21416150 PMC3393103

[62] Bourdetsky D, Schmelzer CE, Admon A 2014 The nature and extent of contributions by defective ribosome products to the HLA peptidome. Proc Natl Acad Sci U S A 111:E1591–E1599. doi:10.1073/pnas.1321902111. 24715725 PMC4000780

[63] Fortier MH, Caron E, Hardy MP, Voisin G, Lemieux S, Perreault C, Thibault P 2008 The MHC class I peptide repertoire is molded by the transcriptome. J Exp Med 205:595–610. doi:10.1084/jem.20071985. 18299400 PMC2275383

[64] Apcher S, Daskalogianni C, Lejeune F, Manoury B, Imhoos G, Heslop L, Fahraeus R 2011 Major source of antigenic peptides for the MHC class I pathway is produced during the pioneer round of mRNA translation. Proc Natl Acad Sci U S A 108:11572–11577. doi:10.1073/pnas.1104104108. 21709220 PMC3136330

[65] Farfan-Arribas DJ, Stern LJ, Rock KL 2012 Using intein catalysis to probe the origin of major histocompatibility complex class I-presented peptides. Proc Natl Acad Sci U S A 109:16998–17003. doi:10.1073/pnas.1210271109. 23027972 PMC3479494

[66] Grant EP, Michalek MT, Goldberg AL, Rock KL 1995 Rate of antigen degradation by the ubiquitin-proteasome pathway influences MHC class I presentation. J Immunol 155:3750–3758. 7561079

[67] Hickman HD, Luis AD, Bardet W, Buchli R, Battson CL, Shearer MH, Jackson KW, Kennedy RC, Hildebrand WH 2003 Cutting edge: class I presentation of host peptides following HIV infection. J Immunol 171:22–26. doi:10.4049/jimmunol.171.1.22. 12816978

[68] Caron E, Vincent K, Fortier MH, Laverdure JP, Bramoulle A, Hardy MP, Voisin G, Roux PP, Lemieux S, Thibault P, Perreault C 2011 The MHC I immunopeptidome conveys to the cell surface an integrative view of cellular regulation. Mol Syst Biol 7:533. doi:10.1038/msb.2011.68. 21952136 PMC3202804

