# Supplementary material for: Early Kinetics of the HLA Class I-Associated Peptidome of MVA.HIVconsv-Infected Cells
Source: J Virol. 2015 Mar 25;89(11):5760–71. doi: 10.1128/JVI.03627-14 (PMC4442425; doi:10.1128/JVI.03627-14)
Supplement: Supplemental material [file supp_89_11_5760__index.html]

Early Kinetics of the HLA Class I-Associated Peptidome of MVA.HIVconsv-Infected Cells — Supplemental material 

# Early Kinetics of the HLA Class I-Associated Peptidome of MVA.HIVconsv-Infected Cells

## Supplemental material

**Files in this Data Supplement:**

- Supplemental file 1 -

  Table S1 (Peptide sequences derived from the MVA vector.)

  Table S2 (Correlations between HLA-associated peptide and protein abundances.)

  PDF, 543K
