# Supplementary material for: Early Kinetics of the HLA Class I-Associated Peptidome of MVA.HIVconsv-Infected Cells
Source: J Virol. 2015 Mar 25;89(11):5760–71. doi: 10.1128/JVI.03627-14 (PMC4442425; doi:10.1128/JVI.03627-14)
Supplement: Supplemental material [file JVI.03627-14_zjv999090414so1.pdf]

Supplementary Table 1

Peptide	Accession	Peaks score (-10lgP)	Mass	ppm	m/z	RT
AIRSKEDVPNFK	160858076	56.07	1402.7568	0.3	468.5930	28.05
ALINL	160857967	16.45	542.3428	-0.4	543.3499	56.46
ALYSYASAK	56404437	44.26	972.4916	1.2	487.2537	32.18
APRSGLSL	160857922	19.37	799.4552	0.1	400.7349	37.19
ARPINGISY	160858004	31.21	989.5294	0.6	495.7723	36.85
ATFTVNIFK	56404712	47.15	1039.5702	0	520.7924	58.13
AVKDVTITK	160858009	20.13	973.5807	-1.2	325.5338	23.33
AVTGGAGFLGR	160858054	58.27	1004.5403	0.1	503.2775	40.20
AVYAVTGGAGFLGR	160858054	63.22	1337.7091	-0.2	669.8617	51.19
FFPPEIINV	160857950	27.80	1074.5750	-0.8	538.2943	72.42
FMYPEFARK	160857950	25.76	1187.5797	-1.3	396.8667	43.86
FPANINDKQIM	160857967	53.24	1289.6438	-0.4	645.8289	46.27
FPITIENAL	56404712	32.62	1016.5542	0.1	509.2844	67.49
FPKNDFVSF	160858076	32.81	1099.5338	1	550.7747	57.14
FPQHVITKDVL	160857944	14.39	1295.7238	-0.1	432.9152	44.71
FTLNHVLALK	160857944	28.39	1154.6812	0.7	385.9012	52.47
FYRPLHFQY	56404712	48.71	1269.6294	-0.1	635.8219	48.46
GKIVIV	160857976	18.85	627.4319	0.6	314.7234	41.03
GMWGGGSSSGVK	160857898	56.41	1108.4971	0.4	555.2560	32.79
GMWGGGSSSGVKSGVNGGVK	160857898	27.28	1806.8683	-0.3	603.2965	33.37
GTITPNAPVGK	160857918	34.49	1053.5818	-0.4	527.7980	28.68
GTNANYFSGK	160858026	36.65	1057.4829	0.2	529.7488	29.33
HIGIPISKK	56404705	31.44	991.6178	0.3	331.5466	27.96
HPTSNSLNAL	160858083	18.30	1052.5250	-0.3	527.2697	35.51
IHTAALVDV	160858054	15.03	937.5233	0.8	469.7693	41.09
IIGPMFSGK	22095787	32.91	948.5103	0.4	475.2626	48.54
IKPPLITL	56404439	16.89	893.5950	0.5	447.8050	59.30
ILNPVASSLK	56404551	22.92	1040.6229	-0.1	521.3187	44.44
INSTSIFSRK	56404712	14.28	1151.6299	0.3	384.8840	38.08
IPGDILSII	160857944	14.84	939.5640	0	470.7893	62.83
IPSPGIML	160858095	24.11	826.4622	0.1	414.2384	62.33
IPVGATLHL	160857950	28.04	919.5491	0.6	460.7821	52.75
ISLRSL	56749684	14.31	687.4279	0.1	344.7213	46.61
IVSGRVFNDK	160857884	48.77	1133.6193	-0.3	567.8167	28.36
IWDNESNVM	160858043	43.34	1106.4702	-0.4	554.2422	48.21
KINMSSGMR	56404709	58.37	1022.5001	0.5	512.2576	24.38
KLFSDISAIGK	160857943	55.62	1177.6707	-0.2	589.8425	46.69
KLNLSPLLTK	56404712	30.24	1125.7122	-0.5	563.8631	47.93
KMIDDYLTRK	56404550	50.70	1281.6750	-0.4	641.8445	34.80
KMIDDYLTRKK	56404550	30.17	1409.7700	0.9	470.9311	31.25
KPNSFTFSF	56404712	28.55	1073.5182	0.3	537.7665	59.93
KQYPAGRPNYVK	81922768	15.92	1419.7622	0	474.2614	21.10
KSNPFITELNNK	160858043	59.68	1403.7408	-0.5	702.8774	41.25
KTIKDFVNK	160857950	38.77	1091.6339	-2.1	546.8231	27.58
KTYTSGGGGMWGGGSSSGVK	160857898	87.78	1859.8472	0.8	930.9316	32.40
KTYTSGGGGMWGGGSSSGVKSGVNGGVK	160857898	81.78	2558.2183	0.5	853.7471	32.97
KVDPVEFVKK	160857910	36.48	1187.6914	0.6	396.9046	33.07
KVFDKSLLY	160857993	34.43	1111.6277	-0.6	556.8208	44.85
KVFDKSLLYK	160857993	51.64	1239.7227	-0.1	414.2481	36.62
KVFFGPIYY	56404712	52.45	1132.5957	-0.1	567.3051	62.78
KVGINLVEK	56404712	20.95	998.6124	0.6	500.3138	34.79
KVNNFTLNH	160857944	60.34	1085.5618	0.2	543.7883	29.44
KVSPPSLGK	160857959	42.65	911.5439	1	456.7797	26.25
KVVDTFISY	160858056	44.96	1070.5648	0.3	536.2899	50.26
KVYEAVLRH	160857944	54.40	1113.6294	-0.1	372.2170	26.16
KVYEILKINSVK	56404441	21.89	1432.8654	-0.1	478.6290	41.58
LKMVTSVIK	160857993	14.09	1017.6256	-1	509.8195	42.50
LVYFSTQQNK	160858056	51.62	1226.6295	-0.2	614.3219	39.37

LYDLQRSAM	160857941	49.82	1095.5382	0.3	548.7766	41.38
LYKSINVEY	160858069	46.85	1127.5862	0.2	564.8005	43.01
MINDDSFTL	160857943	21.37	1054.4641	-0.8	528.2389	59.76
NPSKMVYAL	160857943	21.55	1021.5266	1.4	511.7713	45.89
NVTDIVKAL	160857950	38.17	971.5651	0	486.7899	58.12
NYVDY	160858083	15.47	672.2755	-0.2	673.2827	36.35
NYVDYNII	160858083	18.65	1012.4866	0.6	507.2509	58.46
NYVDYNIIF	160858083	47.73	1159.5549	0.1	580.7848	71.00
NYYIHPIL	160858004	19.61	1031.5439	-2.3	516.7781	56.69
RIISYNPPPK	160857928	54.31	1183.6713	0.4	592.8431	28.76
RLHEILTVK	56404712	52.11	1107.6764	0.3	370.2328	32.10
RLNPCVVRR	160857950	27.54	1111.6396	0.4	371.5540	28.10
RMAEQLVMK	160858054	32.97	1104.5784	0.4	553.2967	32.22
RMLDTSEKYSKGYK	160857967	55.26	1704.8505	0.9	569.2913	24.05
RPILGDKF	56404712	34.37	944.5443	0.4	473.2796	38.69
SIHAKITSYK	160858076	52.58	1146.6396	-0.1	574.3270	23.24
SKEDVPNFK	160858076	39.78	1062.5345	-0.3	532.2744	27.62
SLFKNVRLLK	56404441	35.93	1216.7655	-0.4	609.3898	44.40
SLIDYSRTNK	160858080	21.31	1195.6196	-1	598.8165	31.76
SLRSTII	160857950	17.20	788.4756	0.2	395.2451	40.74
SLRSTIIKK	160857950	28.41	1044.6655	0.5	349.2293	23.72
SLVSLL	160857894	15.00	630.3952	0	631.4025	61.69
SPRIGDQL	160858004	33.93	884.4716	0.5	443.2433	34.99
STYWKIGVQK	56404728	37.58	1208.6553	-0.1	605.3348	39.96
SVFIHGHDGSNK	160857898	19.71	1296.6211	-0.1	433.2143	21.16
SVNEYHMLK	56404436	27.53	1119.5383	-1.2	560.7758	30.78
TLITLILSNK	160857967	22.01	1114.6962	-1.3	558.3546	68.32
TLNHVLALK	160857944	44.16	1007.6127	0.1	504.8137	38.41
TLYSGNSILYK	160858080	49.66	1257.6605	-0.5	629.8372	44.72
TSGGGGMWGGGSSSGVK	160857898	66.52	1467.6412	0.4	734.8282	33.27
TSGGGGMWGGGSSSGVKSGVNGGVK	160857898	40.89	2166.0125	0.3	723.0117	33.97
VIGGVFINK	56404712	56.64	945.5647	-0.2	473.7895	45.64
VLHVTDTNK	160858090	30.05	1025.5505	0.7	513.7829	21.31
VVADLSAHNKLFK	56404441	69.04	1440.8088	-1.2	721.4108	38.16
VVLTGGTGVGK	160857959	43.77	986.5760	-0.7	494.2950	30.57
VVMHINSPFK	160857967	29.01	1170.6219	0.1	391.2146	44.49
YKPVYSYVL	160857910	41.00	1130.6012	0.1	566.3079	56.12
YLYNKYSFK	160858056	32.12	1224.6179	0.2	613.3163	38.62
YRHTIESVY	160857927	42.89	1166.5720	0.3	584.2935	31.93
YYDGNIYDL	160857903	32.04	1134.4869	0	568.2507	57.61

Supplementary Table 2

HLA-associated peptide sequence	Protein of origin	correlation coefficient
EHPVLL	A5A3E0	-1.00
IVSELL	O14744	-1.00
EFTRIL	O14950	-1.00
VVFVIDPGFAK	O43143	-1.00
AIDQLHLEY	O43707	-1.00
ATPLSTLSLK	O60341	-1.00
NRLLEATSY	O60341	-1.00
TVIIKY	O60749	-1.00
LYHGYIYTY	O60880	-1.00
FFGTHETAF	O75475	-1.00
SPKKPVIVF	O75643	-1.00
FANAL	O75874	-1.00
SPGPEKMAL	O75995	-1.00
SVFGGLVNYFK	O95721	-1.00
FAKALANVNIGSL	P05386	-1.00
KLSPPMLLK	P06400	-1.00

KVGEVIVTK	P10809	-1.00
IDLDPETEQVNG	P11586	-1.00
DPETEQVNGLF	P11586	-1.00
LTDITKGVQY	P13639	-1.00
QETSFTKEAY	P13693	-1.00
NAANVGWNNSTFA	P14174	-1.00
AISSFIAYQKK	P14209	-1.00
MARGAALAL	P14209	-1.00
DSEIKIMK	P17980	-1.00
SPMAERSMM	P18583	-1.00
FDKITSRIQK	P23921	-1.00
LTGPVMPVR	P26373	-1.00
ARGPIQIL	P30876	-1.00
IRSSYIRVL	P33992	-1.00
SYIRVL	P33992	-1.00
RTYLPSQVSR	P35606	-1.00
KTEVNSGFFY	P40227	-1.00
QLYDKGLVY	P41252	-1.00
LNGAAL	P42704	-1.00
RLIFSTITSK	P43686	-1.00
DIVHSF	P49327	-1.00
DIVHSF	P49327	-1.00
FRGPSIAL	P49327	-1.00
FRGPSIAL	P49327	-1.00
TIFGKIIRK	P49773	-1.00
LSSLII	P50454	-1.00
YRPPELLL	P50750	-1.00
KLADFGLAR	P50750	-1.00
SMFTAILKK	P50851	-1.00
SVYKASLSLIEK	P52292	-1.00
LRPDMVLEL	P53675	-1.00
AVGITKLL	P55060	-1.00
GVRDVFERPSAK	P55081	-1.00
EEVHDLERKY	P55209	-1.00
LVANFFPKK	P61289	-1.00
VPDSSGPERIL	P61978	-1.00
VPDSSGPERIL	P61978	-1.00
QLYWSHPRK	P62273	-1.00
DVTSRVTYK	P62826	-1.00
LLREAESLIAK	P78371	-1.00
SLAPVNIFK	P78371	-1.00
DKARRILE	P78527	-1.00
LFDANKAEL	Q01082	-1.00
LPRQPPMSL	Q13045	-1.00
LPHAPGVQM	Q13547	-1.00
FPFSDKLGEL	Q14677	-1.00
YIDEQFERY	Q15019	-1.00
SPRGPGQGSGHL	Q15027	-1.00
KLGEFFQTK	Q15029	-1.00
ALWDRGLL	Q15393	-1.00
SMVEFNGK	Q16531	-1.00
AVNAHSNILK	Q16891	-1.00
FRVDYILSV	Q5JWF2	-1.00
IVIKGSEL	Q6P2Q9	-1.00
IVIKGSEL	Q6P2Q9	-1.00
FLDASGAKLDY	Q7L1Q6	-1.00
NIHFM	Q7L1Q6	-1.00
SPTLPAARSSL	Q7Z2W4	-1.00
MPEQIVIHAL	Q8N3U4	-1.00
ALLRVTPFILK	Q8N4H5	-1.00
VPQSGVPAL	Q8WWM7	-1.00

NRPEFITEF	Q8WXH0	-1.00
ALIDPSSGLPNR	Q92530	-1.00
FVDPNGKISL	Q92538	-1.00
RPRPTEATVSL	Q92619	-1.00
SVYGYGVQK	Q96I24	-1.00
YVDRVTEF	Q96JB5	-1.00
KPQTKLLIL	Q99081	-1.00
LPRKPVAGA	Q99627	-1.00
LTQEEF	Q9P016	-1.00
RLVDLPISK	Q9ULT8	-1.00
TFDETVSTY	Q9ULW0	-1.00
GLFQGKTPLRK	Q9ULW0	-1.00
FYMDTSHLF	Q9Y520	-1.00
FYMDTSHLF	Q9Y520	-1.00
YTRNTKGGDAPAAGEDA	P62851	-0.95
YKPPGFSL	Q92621	-0.95
QVIYQL	Q92621	-0.95
FPVPKGVL	A0FGR8	-0.80
RVAPEEHPVL	A5A3E0	-0.80
RVAPEEHPV	A5A3E0	-0.80
FPQYPDKEL	A5YKK6	-0.80
LPDPFTPNL	A5YKK6	-0.80
VVLYPLVAK	O00170	-0.80
SGKTAAFLL	O00571	-0.80
VARPLSTAL	O14561	-0.80
KLISTLIYK	O14980	-0.80
TRVDTILEF	O14980	-0.80
PIFKVAPSK	O15042	-0.80
RIFEPPPPK	O43143	-0.80
SMNANTITK	O43290	-0.80
MPSESAAQSL	O43660	-0.80
FPNKQGYVL	O43684	-0.80
RLYDVPANSMRLK	O43684	-0.80
IYPVNAISF	O43684	-0.80
LFDHAVSKF	O60488	-0.80
LPVALQTRL	O60828	-0.80
GTWSTGLPK	O60828	-0.80
IVAGSLITK	O75400	-0.80
VVLGQFLVLK	O75531	-0.80
RTGKPIAVKL	O75534	-0.80
KLPEEVVKK	O75643	-0.80
DVATFL	O75643	-0.80
RPFGSISRIYL	O75821	-0.80
SPNGTIRNIL	O75874	-0.80
AHPTDVSIS	O95359	-0.80
LRPPLLAL	P05107	-0.80
AKALANVNIGSL	P05386	-0.80
MRYVASYL	P05387	-0.80
ITFPGLHEL	P06239	-0.80
RVKDIGYIFK	P06400	-0.80
RIHPVSTMVK	P07195	-0.80
QLDRISVYY	P07437	-0.80
IRKPYIWEY	P07814	-0.80
FTDVNSILRY	P07814	-0.80
TEVFV	P08575	-0.80
NTDSPLRY	P08865	-0.80
KPLVIIAEDVDGEALSTLVLNR	P10809	-0.80
TVFDAKRLIGR	P11142	-0.80
VALPGVAVSML	P12074	-0.80
VVQDGITLITK	P13010	-0.80
VYGPLPQSF	P13073	-0.80

KKSDPVVSY	P13639	-0.80
YFDPANGKF	P13639	-0.80
AISSFIAYQK	P14209	-0.80
LMYGPPGTGK	P17980	-0.80
VVGSQGMPK	P17987	-0.80
QIFNGTFVK	P18124	-0.80
VPAEPKLAF	P18124	-0.80
EPSAVALEL	P18583	-0.80
SPMADRSMM	P18583	-0.80
QVAQMQLKY	P19623	-0.80
TPEELGLDKV	P20674	-0.80
KTLDEILQEK	P21127	-0.80
LVIPFTIKK	P21333	-0.80
SADFVVEAIGDDVGTLGFSVEGPSQAK	P21333	-0.80
ILNTWISLK	P22234	-0.80
KFSDRTI	P22307	-0.80
KIGGIFAFK	P22307	-0.80
IILGGVKA	P22314	-0.80
IILGGVKA	P22314	-0.80
AIEHTL	P22314	-0.80
AIEHTL	P22314	-0.80
ATTTAAVVGL	P22314	-0.80
ATTTAAVVGL	P22314	-0.80
AANPIQFTL	P23921	-0.80
KTYQDIQNTIK	P24928	-0.80
SLMRVLSEK	P24928	-0.80
VLAAANPVYG	P25205	-0.80
ATFPLSVQK	P26196	-0.80
IPIALSGRDIL	P26196	-0.80
RLFGNILDK	P26358	-0.80
APRPASGPIRP	P26373	-0.80
TLSGWILSK	P27824	-0.80
NKIEKIYIM	P28908	-0.80
IRMPSLPSY	P29401	-0.80
FVYKGGKIYK	P31150	-0.80
GYGGGYGGQSSM	P31943	-0.80
RPAEVGGMQL	P33316	-0.80
SPSSIRSL	P33992	-0.80
IPRSITVLV	P33993	-0.80
RSGGGGGGGLGSGGSIRSS	P35527	-0.80
KVYENYPTY	P35659	-0.80
RMFAPTKTWR	P36578	-0.80
IGILSR	P40939	-0.80
SPTIGKQL	P42285	-0.80
SPTIGKQL	P42285	-0.80
GTKGPSNVFK	P42285	-0.80
GTKGPSNVFK	P42285	-0.80
YLKEAVTTLK	P42704	-0.80
KTYVTP	P46781	-0.80
VRPPVQVYGIEG	P48047	-0.80
FGMQDGSVNMREK	P49247	-0.80
SLAAYY	P49327	-0.80
SLAAYY	P49327	-0.80
SPAPTHNSL	P49327	-0.80
SPAPTHNSL	P49327	-0.80
HPIEGSTTV	P49327	-0.80
HPIEGSTTV	P49327	-0.80
DGKVSV	P49327	-0.80
DGKVSV	P49327	-0.80
AGLLEI	P49368	-0.80
ITDSAGHILY	P49755	-0.80

FPDSGHFNV	P50990	-0.80
IISESF	P50991	-0.80
RPSGPGPEL	P51531	-0.80
LPIVTPAL	P52292	-0.80
IDLIIK	P53618	-0.80
NTDEPPMVF	P54578	-0.80
NTDEPPMVF	P54578	-0.80
APEEHPVLL	P60709	-0.80
RVAPEEHPV	P60709	-0.80
GRDLTDYL	P60709	-0.80
HIFPAL	P61160	-0.80
KLDGSRLIK	P62081	-0.80
HVTQEDFEM	P62195	-0.80
HVTQEDFEM	P62195	-0.80
LPNKVDPLV	P62195	-0.80
LPNKVDPLV	P62195	-0.80
KTVTAMDVVYALK	P62805	-0.80
DVVYALK	P62805	-0.80
KLNNLVLFDK	P62851	-0.80
QIFVKTLTGK	P62979	-0.80
TPQFPDMIL	P63244	-0.80
ATLKWILENK	P78347	-0.80
QTIIA	P78371	-0.80
QNNFSLAMKL	P78527	-0.80
ALFSRIFGK	P84085	-0.80
SLYAHGYLK	P98175	-0.80
LLDQGQLNKY	Q00610	-0.80
FNIEMK	Q00610	-0.80
KIFQEM	Q01082	-0.80
KLRQPFFQK	Q01105	-0.80
AQYSPQQLAGK	Q01581	-0.80
RPTGGVGAV	Q01581	-0.80
IYDEIQQEM	Q01826	-0.80
FTDEEGYGRY	Q12874	-0.80
NYPGLSISL	Q13011	-0.80
LTDDGNKWLY	Q13200	-0.80
FPMAHPPNL	Q13435	-0.80
TNTSVL	Q14008	-0.80
RIYKPLRTR	Q14137	-0.80
KIHEPIMLK	Q14152	-0.80
QRYLVYAI	Q14204	-0.80
FRVPLAIVN	Q14204	-0.80
VLYENPNLK	Q14204	-0.80
SPRLPVGGF	Q14669	-0.80
SPRIATTTA	Q14980	-0.80
AFSILFK	Q14C86	-0.80
FFDHSGTL	Q15005	-0.80
WTPHLYARL	Q15021	-0.80
KAGEVFIHK	Q15233	-0.80
NPAQDFSTL	Q15648	-0.80
NPNQNKNVAL	Q15717	-0.80
VTYNGVDNNK	Q16181	-0.80
ALRDNSTMGYMMAK	Q58FF7	-0.80
PRPPPKPM	Q5JNZ5	-0.80
APRPPPKPM	Q5JNZ5	-0.80
VFDESLNF	Q5SW79	-0.80
YPEELKPVL	Q5UIP0	-0.80
NSIVLLGK	Q6NXT6	-0.80
LTTKPI	Q6P2Q9	-0.80
LTTKPI	Q6P2Q9	-0.80
YRTDMIQAL	Q6P2Q9	-0.80

YRTDMIQAL	Q6P2Q9	-0.80
RTLDAKMPRK	Q6PKG0	-0.80
LPSTRLGTL	Q7KZF4	-0.80
HPQPIDPLL	Q86VP6	-0.80
TLSNQPLLK	Q8N163	-0.80
GPAPVGRFF	Q8TDP1	-0.80
LSLIML	Q8WUM4	-0.80
RTLQKQSVVYGGK	Q8WWY3	-0.80
ALMPQETQALK	Q8WYJ6	-0.80
AKLTVI	Q8WZ42	-0.80
AVVTGL	Q8WZ42	-0.80
EAVESMVK	Q96C01	-0.80
YVDRVTEFL	Q96JB5	-0.80
VDVSIL	Q96JJ3	-0.80
YPENGVVQM	Q96T88	-0.80
NGVVQM	Q96T88	-0.80
AMFSPPVNSGK	Q99081	-0.80
GVSAATFLK	Q99613	-0.80
AESAFSFK	Q99627	-0.80
VERLISSL	Q9BQ52	-0.80
SLVIRSPSLLQSGAK	Q9BQG0	-0.80
PRPPILGY	Q9BXP5	-0.80
IRDNFVIIY	Q9BXS5	-0.80
YRQKQVVIL	Q9BZX2	-0.80
APTPIQALTL	Q9GZR7	-0.80
VPRPVLRAL	Q9GZR7	-0.80
NPRQAAYEM	Q9H0D6	-0.80
FPDTGSLNL	Q9H3P2	-0.80
ATGQWTRFK	Q9H3P2	-0.80
RIYKGVIQAIQK	Q9NQC3	-0.80
PTAPAAGAPL	Q9NQC3	-0.80
LPNAVITRI	Q9NRF9	-0.80
HATSKAKKL	Q9NTJ3	-0.80
KVLDAIIQEK	Q9NTJ3	-0.80
SVTGNAL	Q9NYB0	-0.80
MPEPTVLSL	Q9P0U1	-0.80
VVNSYPLAH	Q9UHI6	-0.80
RPWLEGRHTL	Q9UI10	-0.80
MPRGVVVTL	Q9ULT8	-0.80
AVYGQNDIHHK	Q9UN86	-0.80
MPSVPDFETL	Q9UNS2	-0.80
YPTVDSNSL	Q9UQ35	-0.80
SEKDTKQIL	Q9Y230	-0.80
FLDESRSTQY	Q9Y230	-0.80
ASLVGQTSPK	Q9Y2X3	-0.80
KPMEKLLGL	Q9Y3D3	-0.80
LTGTPGVGK	Q9Y3D8	-0.80
ALRYPMAVGLNK	Q9Y3U8	-0.80
FLDSTGSRLDY	Q9Y6E2	-0.80
SSGAHGEEGSARMWKTLTFF	P12074	-0.77
SVPAGGAVAVSAAPG	P05387	-0.74
SLYASSPGGVYATR	P08670	-0.74
RMEESFSSK	P42167	-0.74
RIGNFIVKK	Q00796	-0.74
LSIRGF	Q15631	-0.74
LPVFDKEEL	P54687	-0.63
GFAGDDAPR	A5A3E0	-0.60
NSDGYGGNY	O00571	-0.60
MRYVASYLL	P05387	-0.60
YPIVPLDGKGTL	P06239	-0.60
GVYATRSSAVR	P08670	-0.60

HIHKSLIGK	P0C0S5	-0.60
HIHKSLIGK	P0C0S5	-0.60
NPIITKL	P11142	-0.60
YPVNSVNIL	P17987	-0.60
TILRIDDLIK	P17987	-0.60
FPESSAIEAL	P18583	-0.60
VVGVGMTK	P22307	-0.60
YGVLRF	P23396	-0.60
ESAEAQLL	P23921	-0.60
AIPTTGRGSSGVGL	P25205	-0.60
LAINL	P26196	-0.60
QLRGPVKPTGGPGGGGTQTQQQMNQLK	P26196	-0.60
IVPDIAVGTK	P26599	-0.60
SIFAGQNDPLK	P27816	-0.60
SRPENAIIY	P29401	-0.60
SPIGRDGKL	P30876	-0.60
SPQEARNML	P33240	-0.60
SPIISKPK	P34932	-0.60
DGIPPPY	P40429	-0.60
LVRPPVQVYGIEGRYATAL	P48047	-0.60
VRPPVQVY	P48047	-0.60
IINSSITTK	P49368	-0.60
IPYGERITL	P49750	-0.60
AVFPSLLTNPK	P52292	-0.60
VFPSL	P52292	-0.60
LEIVPN	P52597	-0.60
ARIPFYGSY	P61011	-0.60
MENGIVRNW	P61160	-0.60
APAPPKAEA	P62750	-0.60
NYQPPTVVPGGDLA	P68366	-0.60
NPRQINWTVL	P83731	-0.60
ALFAKVAATK	Q01826	-0.60
YPDEEAIQTL	Q01826	-0.60
AMYSRKAMYK	Q02878	-0.60
DHIPIL	Q06210	-0.60
KVYNIQIRY	Q13094	-0.60
IRPDNMSEY	Q13547	-0.60
DPDSKPFSL	Q14141	-0.60
NRVPAGNWVL	Q15029	-0.60
RPKPSSSPVIF	Q15366	-0.60
SSLRVL	Q15393	-0.60
YKNIVL	Q16666	-0.60
YKIAFPYLY	Q6P2Q9	-0.60
YKIAFPYLY	Q6P2Q9	-0.60
GNAFHL	Q6P2Q9	-0.60
GNAFHL	Q6P2Q9	-0.60
HLSEPLGGK	Q86X76	-0.60
ARPPGYEF	Q8IU81	-0.60
ARPPGYEF	Q8IU81	-0.60
YPEPAPAAL	Q8IU81	-0.60
YPEPAPAAL	Q8IU81	-0.60
RTVYVYLEK	Q8N3U4	-0.60
ATYYGAFIKK	Q8N4C8	-0.60
YGNIL	Q8NFH5	-0.60
TQTLAI	Q8WYJ6	-0.60
SLVLTI	Q96JB5	-0.60
LRILDI	Q99986	-0.60
VIRSPSLLQSGAK	Q9BQG0	-0.60
LPSDIAAEA	Q9GZR7	-0.60
LSLEIL	Q9UHB9	-0.60
FPEEAVLQQAF	Q9UPN7	-0.60

AIALTM	Q9Y262	-0.60
KTYTSGGGGMWGGGSSSGVKSGVNGGVK	160857898|emb|CAM58186.1|	-0.40
KTYTSGGGGMWGGGSSSGVK	160857898|emb|CAM58186.1|	-0.40
FRPPNPWTM	A5YKK6	-0.40
LPDPFTPNLKV	A5YKK6	-0.40
MPEPDAQRFF	O14757	-0.40
ALKNPPINTK	O15511	-0.40
ISAGLPPLK	O43143	-0.40
EIVKSPSDPK	O43847	-0.40
HLISPLIQK	O43847	-0.40
QPITPGPSI	O60341	-0.40
FWDPSVNLF	O60341	-0.40
FWDPSVNL	O60341	-0.40
VPNQKRLTLL	O60488	-0.40
AISWPLLQK	O75717	-0.40
VFDPHGTL	O95347	-0.40
WGDAGAEYVVESTGVFTTMEK	P04406	-0.40
KIADFGLAR	P06239	-0.40
GLTSVINQK	P07195	-0.40
LVDLEPGTM	P07437	-0.40
TVWNKPTVK	P07602	-0.40
GVHGGLINK	P07737	-0.40
DNMGLVLPPRVACVQVVI	P07814	-0.40
STYPAQNGQVK	P08575	-0.40
LIKTVETRDGQVINETSQ	P08670	-0.40
PETEQVNGLF	P11586	-0.40
IADMGHLKY	P12004	-0.40
GLYQGFSVSVQGIIIYR	P12236	-0.40
KVNPIQGLASK	P13073	-0.40
DAINDANLL	P14324	-0.40
AVFLGLARK	P14324	-0.40
IPAPAEKTPV	P16402	-0.40
FPESSAIEALRL	P18583	-0.40
LPKTTALEL	P18583	-0.40
ALATALGDK	P20700	-0.40
AEIVEGENHTY	P21333	-0.40
ILSGPFVQK	P22102	-0.40
SIDGRVVEPK	P22626	-0.40
LVGDVGQTV	P23528	-0.40
SLLSQMLHY	P24941	-0.40
ALAIKVLNK	P25789	-0.40
SAAAVSMLK	P25789	-0.40
FPRKPSAPK	P26373	-0.40
ALIQQATTVK	P32969	-0.40
SGGGGGGGLGSGGSIR	P35527	-0.40
VNVEINVAPGKD	P35527	-0.40
KIFKNFKEK	P35606	-0.40
KLKAWNDIKK	P36578	-0.40
NPRNFSDNQL	P37802	-0.40
GLFSGDPNWFPK	P37802	-0.40
IGEKAVLLK	P42696	-0.40
KLIGEYGLRNK	P46781	-0.40
GSYNKVFLAK	P48556	-0.40
TAVENMPSL	P49327	-0.40
TAVENMPSL	P49327	-0.40
HPIEGSTTVF	P49327	-0.40
HPIEGSTTVF	P49327	-0.40
LPKTGTVSL	P49327	-0.40
LPKTGTVSL	P49327	-0.40
SALQSV	P49792	-0.40
KLFPGSPAIYK	P49792	-0.40

HPFPGPGLAI	P49915	-0.40
NKPIEEII	P50395	-0.40
VVMRDPQTK	P51991	-0.40
SVKPGAHLTVK	P51991	-0.40
KVDGRVVEPK	P51991	-0.40
IPNEIIHAL	P52272	-0.40
LRIFALLAGK	P52948	-0.40
GVRDVFERPSAKK	P55081	-0.40
QNPQILAAL	P55209	-0.40
APDKHHITPLL	P58546	-0.40
APEEHPVL	P60709	-0.40
GFAGDDAPR	P60709	-0.40
YEGYALPH	P60709	-0.40
RLYQVEYAFK	P60900	-0.40
KMGGIKGLFK	P61011	-0.40
KLLDAYLLY	P61803	-0.40
KLLEPVLLLGK	P62249	-0.40
MIEPRTLQY	P62249	-0.40
KLYDIDVAK	P62750	-0.40
ALKRQGRTLYG	P62805	-0.40
IPHMDIEAL	P62906	-0.40
VIATKVLGT	P67809	-0.40
ILNHPGQI	P68104	-0.40
KLEDGPKFLK	P68104	-0.40
VIDSAELQAY	P78527	-0.40
LPRPPPPEM	Q00325	-0.40
VLNGNPLLH	Q13045	-0.40
VYPERSTSY	Q13242	-0.40
LRTFSW	Q13283	-0.40
AVYGQKEIHRK	Q13283	-0.40
MQTFVL	Q13283	-0.40
ATTHEIMGPK	Q13492	-0.40
SIYRPSKNLDK	Q13573	-0.40
TAALGLL	Q14257	-0.40
LPSQKTASL	Q14684	-0.40
LIAPIFLHR	Q15022	-0.40
KSYPSLTTK	Q15029	-0.40
MLFPGSIALK	Q15691	-0.40
AFPPSVRALL	Q5VT52	-0.40
ALFAKPLKGK	Q6IQ49	-0.40
VYPQYVIEY	Q7Z2W4	-0.40
APYSRPKQL	Q86V81	-0.40
VPNKGYSSL	Q8IW50	-0.40
RIRQLPVGK	Q8IY81	-0.40
DVQNPL	Q8TEM1	-0.40
KIIEDKTFGLK	Q8WU90	-0.40
QIFKPIISK	Q92769	-0.40
VPVPPNVAF	Q92888	-0.40
ALNHKVLHY	Q96JJ3	-0.40
SPEQSRNVL	Q96JM3	-0.40
SYNDYVREF	Q96MU7	-0.40
YRGQPGNAY	Q96PK6	-0.40
RVKYGTVFK	Q96ST3	-0.40
HAVGPSTSL	Q99081	-0.40
SIYPHGSTDK	Q99436	-0.40
RTIAPIIGR	Q99459	-0.40
KIKEIAVTVK	Q99832	-0.40
EVSGAGSSPVSGGVN	Q9BQ61	-0.40
TPRPTAAEL	Q9BSJ8	-0.40
ALYPHVLVK	Q9BUJ2	-0.40
SPAKNPSSL	Q9GZR7	-0.40

KLPNTVLGK	Q9H4M9	-0.40
MMDPNSTQRY	Q9HAV4	-0.40
LLVKNL	Q9HB71	-0.40
HPDKDVRLL	Q9NTI5	-0.40
TVSNASSSSNPSSPGRIK	Q9NTI5	-0.40
AVIGADSVTLK	Q9NYH9	-0.40
PVRAPAVAV	Q9P2E9	-0.40
FLANAL	Q9UHI6	-0.40
AVLIQLSPP	Q9UKV3	-0.40
IFDQSGTYL	Q9UMS4	-0.40
APSDNRVTSF	Q9UNZ2	-0.40
LPIDKEEVSL	Q9UQE7	-0.40
TPYSEKDTKQIL	Q9Y230	-0.40
ALYRLAQKK	Q9Y285	-0.40
YMDTSHLF	Q9Y520	-0.40
YMDTSHLF	Q9Y520	-0.40
VPKVVGSAVG	Q9Y5A9	-0.40
SPRVPGGSP	Q9Y6G9	-0.40
KVIDRILYK	O75533	-0.32
ALEAVKRL	O95347	-0.32
KVLSILLKH	P19838	-0.21
VLSVELPGL	A0FGR8	-0.20
RPKDPNNLL	O00232	-0.20
MPQSGTGVSV	O00487	-0.20
YPEPFLIKL	O15042	-0.20
IPIHADPRL	O15160	-0.20
VPNQKRLTL	O60488	-0.20
KLFDHAVSKF	O60488	-0.20
IVPGVEGPI	O60841	-0.20
RPDRLVN	O75534	-0.20
EVQPGAPPPPL	O95359	-0.20
MPRTGNGPMSV	O95793	-0.20
SVASAAAVLSH	P04844	-0.20
VPAGGAVAV	P05387	-0.20
RLRSSVPGVR	P08670	-0.20
QIPELPDPSL	P09884	-0.20
LPDGQVITI	P0CG38	-0.20
ATEDPWKTNY	P10768	-0.20
DMAIATGGAVFGEEGLTLNLEDVQPHDLGK	P10809	-0.20
IDLDPETEQVNGLF	P11586	-0.20
NPAENFRVL	P13010	-0.20
VVAGFQWATK	P13639	-0.20
RVRVSGQGL	P21333	-0.20
LPNGLVIASL	P22695	-0.20
MIYASSKDAIKK	P23528	-0.20
TPQSNRPVM	P24928	-0.20
VPNVHGAL	P26599	-0.20
HASDRIIAL	P29401	-0.20
VLYNGFTGR	P30876	-0.20
FGLALAVAGGVVNSALYNVDAGHR	P35232	-0.20
SPNSKVNTL	P40939	-0.20
HPADKDYGL	P41236	-0.20
MPAPEEIVEEL	P46013	-0.20
IPRDPSQQEL	P49327	-0.20
IPRDPSQQEL	P49327	-0.20
TKGPVYGM	P49792	-0.20
EVLTTL	P55036	-0.20
SFVLEF	P55209	-0.20
NSAANASATTTEPLPEKTQESL	P55327	-0.20
YFDSGDYNM	P56211	-0.20
VPPVQVSPLIKLGRYSAL	P56385	-0.20

FLGVAYGATRY	P56385	-0.20
AMFNIRNIGK	P61247	-0.20
LVFPSEIVGK	P62081	-0.20
RPDGEKKAYVRL	P62750	-0.20
DVVYAL	P62805	-0.20
RVKLPSGSK	P62917	-0.20
RPTDKPLRL	P68104	-0.20
RLPLISGFYK	P78527	-0.20
FPQYSPKM	P78527	-0.20
FRAFLGEL	P78527	-0.20
YPDAKMDPM	P78527	-0.20
IYDSVKVYF	Q00325	-0.20
LPTSVVTIT	Q01082	-0.20
WPKDVGIVAL	Q01581	-0.20
RIFAPNHVVAK	Q02543	-0.20
APIQGNREEL	Q13435	-0.20
AVYNSWKNNK	Q14562	-0.20
VPKTTGEGTSL	Q14566	-0.20
LPNFGSHVL	Q14C86	-0.20
QPNPLPLRL	Q15154	-0.20
VLNTSLKEK	Q16666	-0.20
KLRESTPGDSPSTVNK	Q5T6F2	-0.20
RVSYQKLLK	Q6P2Q9	-0.20
RVSYQKLLK	Q6P2Q9	-0.20
VPRVPTHTL	Q8IWZ3	-0.20
RVTPFILKK	Q8N4H5	-0.20
RVTPFILK	Q8N4H5	-0.20
VMPPDQII	Q8WZ42	-0.20
GLECVVRNL	Q8WZ42	-0.20
PIIDGGSPII	Q8WZ42	-0.20
KVLFPLLTK	Q92538	-0.20
SPGPSRPGL	Q92888	-0.20
RLSESQLSFR	Q96PK6	-0.20
ARYSGSYNDY	Q96PK6	-0.20
YRAQPSVSL	Q96PK6	-0.20
KEPQPEQPQPS	Q9GZR7	-0.20
DLFKDIQSL	Q9H4M9	-0.20
IPQKQREITL	Q9NR30	-0.20
IPVESEVNL	Q9NRZ9	-0.20
FTDEESRVF	Q9NYF8	-0.20
SIREAGGAF	Q9UII2	-0.20
RPKNLMQTL	Q9Y2D5	-0.20
KVGENVLPPK	Q9Y520	-0.20
KVGENVLPPK	Q9Y520	-0.20
TPSEPHPVL	Q9Y5A9	-0.20
VPKVVGSAV	Q9Y5A9	-0.20
SPRIGDQL	160858004|emb|CAM58292.1|	0.00
RPHRGILAV	B0I1T2	0.00
APKRPPSAF	B2RPK0	0.00
SPGIWQLD	HIVconsv	0.00
SPRQPSGGQI	O14497	0.00
VSDPSSPQYGK	O14773	0.00
WRPPPLNPY	O15042	0.00
IGNLNTLVVK	O60812	0.00
LPSHVVTML	O75390	0.00
SESNFARAY	O75390	0.00
LLNGKVGSFK	O75995	0.00
QVTQPTVGMNFKTPRGPV	P0CW22	0.00
ALNFAFKDK	P12236	0.00
IPIKKSDPV	P13639	0.00
VSYPKSLSK	P17252	0.00

FYQEHPDL	P17844	0.00
AMSERALAQK	P18583	0.00
HIGGSPFK	P21333	0.00
FVDPAQITM	P23921	0.00
FPTGDSKVVL	P24928	0.00
RVAPRSGLAAK	P33316	0.00
ILQKNVPIL	P35221	0.00
SRSGGGGGGGLGSGGSIRSSY	P35527	0.00
NEDNGIIKAF	P36578	0.00
ALFGQPPFK	P41252	0.00
GEKAVLLK	P42696	0.00
QVFKLGLAK	P46781	0.00
LSYNTASNK	P49207	0.00
LPRLTPPVL	P50990	0.00
TADPLDYRL	P52948	0.00
RVRPKALQTTGTAK	P52948	0.00
FPQKWPDLL	P55060	0.00
RILPKPTRK	P62081	0.00
QIYAIRQSISK	P62249	0.00
ISFPATGCQK	P62753	0.00
RVKLPSGSKK	P62917	0.00
PPAENSSAPEAEQGGAE	P67809	0.00
KLIKDGLIIRK	P84098	0.00
GWDEALLTM	Q00688	0.00
FFDTNTSVL	Q14008	0.00
AVFPVAPTSSK	Q14157	0.00
SPRLPVGGFRSL	Q14669	0.00
ATWATKELRKLK	Q14974	0.00
RLRDPAQQVRK	Q15021	0.00
YSDPSTGEPATY	Q15149	0.00
AIKAMAK	Q6IS14	0.00
VAIKAMAK	Q6IS14	0.00
VLNSIVLLGK	Q6NXT6	0.00
SPEAKLQL	Q8WXH0	0.00
LKATVL	Q8WXH0	0.00
VATLKI	Q8WZ42	0.00
KKDGTLLK	Q8WZ42	0.00
APAGTTSSRVL	Q96JP5	0.00
KVLALQARK	Q96SB4	0.00
VKTDTVLIL	Q9HB71	0.00
AVFGPDGTLLAK	Q9NQR4	0.00
RLAGTSGSDKGLSGK	Q9P016	0.00
AIFKVLNEK	Q9Y2X3	0.00
SVFIHGHDGSNK	160857898|emb|CAM58186.1|	0.20
GMWGGGSSSGVKSGVNGGVK	160857898|emb|CAM58186.1|	0.20
GMWGGGSSSGVK	160857898|emb|CAM58186.1|	0.20
APEEHPVL	A5A3E0	0.20
KTYIPPKGETKK	B2RPK0	0.20
LDTVVFK	O00487	0.20
FPIPGEPGFPL	O15145	0.20
LKNPPINTK	O15511	0.20
FPLDPQLAKMVI	O43143	0.20
KPVSLGQAL	O60341	0.20
GLTDVILYH	O60506	0.20
SVDPTQTEY	O75534	0.20
VTTSTRTYSLG	P08670	0.20
QELNDRFANY	P08670	0.20
VVMRDPASK	P22626	0.20
NPNLRKNVL	P23193	0.20
AQAESLRYK	P23396	0.20
MIYASSKDAIK	P23528	0.20

RPAANPIQF	P23921	0.20
KPKTPSLTV	P24928	0.20
AGRGFSLEEL	P26373	0.20
TPEGHAVGL	P30876	0.20
ELVSITANK	P34897	0.20
LPAGQSVLL	P35232	0.20
KLPKQPVIVK	P46776	0.20
KERFSPL	P48047	0.20
FFNTPK	P48556	0.20
PRGTPLI	P49327	0.20
PRGTPLI	P49327	0.20
IWEPLAVKL	P49368	0.20
VTQPVPLANK	P49750	0.20
TPKPVSIAT	P49790	0.20
GPLYKNGSL	P49792	0.20
SFDSALQSV	P49792	0.20
NPISTVTEL	P50991	0.20
GPNERIGMV	P52292	0.20
RSYSDPPLK	P52597	0.20
EEADGGLKSW	P52907	0.20
FNDTFVHV	P62263	0.20
APRPSTGPHKL	P62701	0.20
LPRGKGIRL	P62701	0.20
ALKRQGRTLYGFGG	P62805	0.20
DPNLEFVAM	P62826	0.20
RIHGVGFKK	P62899	0.20
RLNKAVWAK	P62899	0.20
TLSSKLLTH	P78371	0.20
TPLPEDNSM	P78527	0.20
VGVTKVI	Q08211	0.20
YFDPQYFEF	Q13347	0.20
GPRTAALGLL	Q14257	0.20
RIIEKVIHR	Q14566	0.20
IVGDPSTAK	Q14566	0.20
RLSDVLKRK	Q14669	0.20
RTGPPMGSRF	Q15056	0.20
TYDPETQEF	Q15067	0.20
AFDLDVVKL	Q15459	0.20
TPTQGSVL	Q16531	0.20
FVYEPKEQK	Q16666	0.20
ALIEELLLYK	Q5VT52	0.20
VYDVVELKF	Q8N684	0.20
PVNVPISQK	Q8WUM4	0.20
VRPPIRSH	Q8WWK9	0.20
ILQANKAT	Q96GA3	0.20
YPHGSTDKL	Q99436	0.20
VYDLLKTNL	Q99460	0.20
TTTSGVKPGTAP	Q9H4A3	0.20
NPIEEISVL	Q9NTJ3	0.20
VLYDRVLKY	Q9UHB9	0.20
AYHELAQVY	Q9UNS2	0.20
ALTPPPI	Q9UPN7	0.20
RLRNDGNAIAK	160858004|emb|CAM58292.1|	0.21
RPKLIGEEL	P54252	0.21
YVDQAELEKY	Q01581	0.21
KVLSSLVTLNK	Q14155	0.21
RLEETQALLRKK	Q14203	0.26
KLNIRPLLR	Q15029	0.26
YRHTIESVYF	160857927|emb|CAM58215.1|	0.40
LYDLQRSAM	160857941|emb|CAM58229.1|	0.40
MINDDSFTL	160857943|emb|CAM58231.1|	0.40

KLFSDISAIGK	160857943|emb|CAM58231.1|	0.40
ARPINGISY	160858004|emb|CAM58292.1|	0.40
AVTGGAGFLGR	56404442	0.40
RMAEQLVMK	56404442	0.40
AVYAVTGGAGFLGR	56404442	0.40
KINMSSGMR	56404709	0.40
VNKITPQLK	56404709	0.40
LPIATQEL	A0FGR8	0.40
APFLRNVEL	A5YKK6	0.40
TPRDLVLRSL	B0I1T2	0.40
SPIETVPVKL	HIVconsv	0.40
YTRPTPVQK	O00571	0.40
APAPVQPPM	O14497	0.40
ALLDGSNVVFK	O15212	0.40
HPYPPGPGVAL	O43660	0.40
AVALPLQTK	O43660	0.40
AVYGMLNLTPK	O43776	0.40
KVSQVIMEK	O75475	0.40
VLGQFLVLK	O75531	0.40
GNKVSAEKVNK	O75534	0.40
SLLGKDVLFLK	P00558	0.40
RPFPKLRIL	P04818	0.40
NPRDLPLMAL	P04818	0.40
ERQLLAPGN	P06239	0.40
TVNPKESILK	P06400	0.40
YLSPVRSPK	P06400	0.40
LPVPAFNVI	P06733	0.40
TIAPALVSK	P06733	0.40
RVFIGNLNTLVVK	P07910	0.40
LLQDSVDFSLADAINTEFK	P08670	0.40
RPSSSRSYVTT	P08670	0.40
GPPRLLLLPL	P09564	0.40
SILRNPVTNK	P09661	0.40
KVCNPIITKL	P11142	0.40
SPVVRVAV	P13639	0.40
KPIQRTIL	P13639	0.40
AVQRTLLEK	P14209	0.40
QPRYPVNSVNIL	P17987	0.40
ALIARSLGK	P18124	0.40
QPASFAVSL	P21333	0.40
VVIQDPMGQK	P21333	0.40
SPVSRIGL	P22695	0.40
AIAQAESLRYK	P23396	0.40
RIRELTAVVQK	P23396	0.40
LPKSPPYTAF	P23588	0.40
KTHAVLVALK	P25786	0.40
APSRNGMVL	P26373	0.40
SPNQARAQAAL	P26599	0.40
YFSKGTLK	P27694	0.40
TGGAPRILA	P27708	0.40
HPQPGAVEL	P27708	0.40
LFDPMTGTF	P29083	0.40
VPRAAFLSPLLP	P30040	0.40
VVYPKVIER	P30566	0.40
SPTENSFTL	P31350	0.40
IVNENLVERF	P31350	0.40
RVLPSITTEILK	P35232	0.40
TPNEERNVM	P35606	0.40
RLKSIKNIQK	P36542	0.40
KLNPYAKTMR	P36578	0.40
EDNGIIKAF	P36578	0.40

ALPALVMSK	P36578	0.40
TPFKGGTL	P42166	0.40
SLFIGEKAVLLK	P42696	0.40
GTGDVKLLK	P43487	0.40
NKVFLAK	P48556	0.40
APQAPPPPPK	P48634	0.40
GPRGSGPPM	P48634	0.40
HVIETLIGKK	P48643	0.40
RLSYNTASNK	P49207	0.40
VPVAKTAEL	P49790	0.40
KVTEGSFVYK	P50395	0.40
RLYGPSSVSFADDFVRSSK	P50454	0.40
TAYGPNGMNK	P50990	0.40
APNAGSRMTQTV	P51659	0.40
RLKPTNPAAQK	P52948	0.40
MPITEVFKEEM	P53396	0.40
KLYRPGSVAYVSR	P53396	0.40
KLYRPGSVAY	P53396	0.40
ALANVSIEK	P53618	0.40
AIRNAMGSL	P55036	0.40
LPVPDPILL	P61289	0.40
EFDNAGAMM	P62136	0.40
RLARYYKTK	P62277	0.40
RLSNIFVIGK	P62701	0.40
GEKKAYVRL	P62750	0.40
VLRDNIQGITK	P62805	0.40
RILGPGLNK	P62906	0.40
VVFRDPYRFK	P62917	0.40
IPVWDQEFL	P63208	0.40
ATYAPVISAEK	P68366	0.40
GLYQRAFQH	P78527	0.40
ALAGHQLIR	P78527	0.40
DLLIKL	P78527	0.40
KLATTILQH	P78527	0.40
STFDTQITK	P78527	0.40
NPASKVIAL	Q00610	0.40
KPRDVSSVEL	Q01082	0.40
AARPATSTL	Q04637	0.40
AARPATSTL	Q04637	0.40
NFDDYTVNL	Q06210	0.40
GPLPPHLAL	Q08211	0.40
KYPSPFFVF	Q08211	0.40
VFDPVPVGV	Q08211	0.40
KPHNPGFGM	Q12906	0.40
AVLQSPGLSGK	Q13045	0.40
ATATQLLQH	Q13188	0.40
RTFSWASVTSK	Q13283	0.40
QEDLRTFSW	Q13283	0.40
RPRGPPPPP	Q13435	0.40
KKPQVVQALQ	Q14008	0.40
GPRTAALGL	Q14257	0.40
TWDEYNIQM	Q14257	0.40
HLEDIVRQK	Q14669	0.40
RTTQIINITMTK	Q14980	0.40
APRKVLGSSTSA	Q15004	0.40
SPRGPGQGSGHLAI	Q15027	0.40
EAIAEL	Q15149	0.40
FPHTTPSM	Q15366	0.40
GPRSSLRVL	Q15393	0.40
LPYLPSGESL	Q16186	0.40
RLGDSQLVK	Q16531	0.40

AQALRDNSTMGYMMAK	Q58FF7	0.40
APRQPGLMAQ	Q5T1J5	0.40
APRQPGLMA	Q5T1J5	0.40
AVAIKAMAK	Q6IS14	0.40
GTYHGMTLK	Q6P1J9	0.40
RLKEAYSVK	Q6P2Q9	0.40
RLKEAYSVK	Q6P2Q9	0.40
AVREQLLAL	Q8IU81	0.40
AVREQLLAL	Q8IU81	0.40
TVDLPKSPK	Q8N1K5	0.40
TVDLPKSPK	Q8N1K5	0.40
VLSTLK	Q8N3U4	0.40
NFDRSSSTF	Q8N3U4	0.40
VSFPIGIYK	Q8TCA0	0.40
RLSEWKAGK	Q8WWK9	0.40
SPASPKISL	Q8WWM7	0.40
EFEFVL	Q8WXH0	0.40
VLVLDKPGPPR	Q8WZ42	0.40
MYGVGEPVQASPIT	Q8WZ42	0.40
NPNMRLSQL	Q96CT7	0.40
APVSGPRL	Q96GA3	0.40
RPVNLMGTSL	Q96JB5	0.40
KFKFPGRQK	Q96L21	0.40
FPEADKVRTM	Q99613	0.40
VWDLFPEADKV	Q99613	0.40
LLDIQSSGRAK	Q99613	0.40
ALASRFLKK	Q99613	0.40
APREALAQTV	Q99829	0.40
VLTLPLMLK	Q99829	0.40
FPISEETIKL	Q9BQ39	0.40
HVLEAQDLIAK	Q9BSJ8	0.40
KASEVFLQR	Q9H0A0	0.40
GLFNRIIRK	Q9H0A0	0.40
FPSEITDTV	Q9H4A3	0.40
ATQGAKIVSL	Q9NQR4	0.40
SRAASVFVL	Q9NRF9	0.40
VVYAPLSKK	Q9NRZ9	0.40
HLADILSSK	Q9UHI6	0.40
RIRDGDFVVLK	Q9UJA5	0.40
SIFDGRVVAK	Q9UM00	0.40
MVTKNDVM	Q9UQE7	0.40
KLGGVIKEK	Q9Y2X3	0.40
TLGKELASK	Q9Y3D8	0.40
FPTVQHQEL	Q9Y520	0.40
FPTVQHQEL	Q9Y520	0.40
SPRSPPSSSEIFTPA	C9JLW8	0.60
AMLDTVVFK	O00487	0.60
RMEEMGVQGGRAK	O15234	0.60
LPLAHVLEL	O60488	0.60
IVYEKIMEH	O75643	0.60
KLNWTGTSK	O95433	0.60
ALFLTLTTK	P00403	0.60
APVYLAAVL	P0C0S8	0.60
FPWGDGNHTL	P12074	0.60
IPLSKIKTL	P13010	0.60
RVFSGLVSTGLK	P13639	0.60
VFDEAIRAV	P15153	0.60
VFDEAIRAVL	P15153	0.60
AASKERSGVSL	P16402	0.60
RLLDSEIKIMK	P17980	0.60
SPEPSKIML	P18583	0.60

LDPIQKL	P18859	0.60
YPFESAEAQL	P23921	0.60
YPFESAEAQLL	P23921	0.60
APRAIPTTGRGSSGVGL	P25205	0.60
ERLGYPVL	P27708	0.60
GTTAVLTQTVK	P40306	0.60
SENLLGKQF	P46013	0.60
VMHTPPVLKK	P46013	0.60
VRSGYYKVL	P46776	0.60
AAAPASVPAQAPK	P47914	0.60
HPLGDIVAF	P49327	0.60
HPLGDIVAF	P49327	0.60
APHALLQAVL	P49327	0.60
APHALLQAVL	P49327	0.60
GLYYIHRNK	P50750	0.60
YPQDNMHQM	P51532	0.60
IPMEFVNKM	P53396	0.60
PLANISQI	P53675	0.60
MPLNVADLI	P60842	0.60
MPSDVLEVT	P60842	0.60
IYDIFQKL	P60842	0.60
SLAGGIIGVK	P61978	0.60
SLAGGIIGVK	P61978	0.60
LLYGPPGTGK	P62195	0.60
LLYGPPGTGK	P62195	0.60
RIDKPILK	P62917	0.60
ALKVPTTEK	Q04637	0.60
ALKVPTTEK	Q04637	0.60
AIFKPVMSK	Q13547	0.60
TPESVL	Q14152	0.60
SPAAKSPSAQL	Q14203	0.60
LPRGSIPRSL	Q14566	0.60
GVDLPSVEA	Q15149	0.60
PGTAFELL	Q15149	0.60
LPVQPENAL	Q5JTV8	0.60
LLAEKVLAGK	Q5JWF2	0.60
QLFEVKVFK	Q5TBB1	0.60
SRFGNAFHL	Q6P2Q9	0.60
SRFGNAFHL	Q6P2Q9	0.60
FPNIVIKGSEL	Q6P2Q9	0.60
FPNIVIKGSEL	Q6P2Q9	0.60
KTFEGNLTTK	Q6P2Q9	0.60
KTFEGNLTTK	Q6P2Q9	0.60
HPNGNITEL	Q7KZF4	0.60
SVFNHAIRH	Q7L576	0.60
FPLSQPFSL	Q8N163	0.60
KVNIIPVIGK	Q8WYJ6	0.60
LRLFVPIKGRPTPE	Q8WZ42	0.60
KMKEALLSIGK	Q96C01	0.60
SYRDSYESY	Q96E39	0.60
AVMAESAFSFK	Q99627	0.60
DVVHPAAK	Q99832	0.60
ILYQTVTGLK	Q9BRS2	0.60
KLFDKLLEY	Q9BZZ5	0.60
ALSIKL	Q9NTJ3	0.60
GVYSNMGNYK	Q9NY33	0.60
RTDDKVIRFK	Q9NZB2	0.60
VISQEPFVPK	Q9ULW0	0.60
TPVAGSQSL	Q9UQ35	0.60
RLWQTVVGK	Q9Y3F4	0.60
RIPGPGTPVK	Q9Y3P8	0.60

MRPLDIVEL	Q9Y605	0.60
RVLSTPDLEVRKK	P53618	0.63
KQNLVIMGK	P00374	0.74
IVLELIPLK	P00403	0.74
PLSTPV	P18583	0.74
STDTSGYGQSSY	P35637	0.74
KLMTVRIVK	P46782	0.74
RVYFGMQDGSVNMREK	P49247	0.74
VYDLSIRGF	Q15631	0.74
RPSLRLQLL	Q7L2H7	0.74
SPRLSQTFL	Q92608	0.74
KVLDKLLLY	Q9BXP5	0.74
KVNIIPIIAK	Q9NVA2	0.74
TSGGGGMWGGGSSSGVK	160857898|emb|CAM58186.1|	0.80
TSGGGGMWGGGSSSGVKSGVNGGVK	160857898|emb|CAM58186.1|	0.80
LVYFSTQQNK	160858056|emb|CAM58344.1|	0.80
KVVDTFISY	160858056|emb|CAM58344.1|	0.80
KMIDDYLTRKK	56404550	0.80
NRWEFASF	56404550	0.80
KMIDDYLTRK	56404550	0.80
APEEHPVLL	A5A3E0	0.80
FPISPIETVPVKL	HIVconsv	0.80
AIFQSSMTK	HIVconsv	0.80
GRNDMTYNY	O14497	0.80
NKADVILKY	O14602	0.80
KLLGPVLVK	O15212	0.80
MLISAGLPPLK	O43143	0.80
TLADILLYY	O43324	0.80
ATFKSFEDRVGTIK	O43399	0.80
CRFPVEIKS	O43776	0.80
STSQTFIYK	O60341	0.80
RVKTNLPIFK	O60493	0.80
LPRAVNTQAL	O60749	0.80
RPYPSPGAVL	O60828	0.80
VFDPIGHF	O60885	0.80
QPRGPDNSM	O75534	0.80
QTYVGITEK	O75643	0.80
MPETYQARL	O75792	0.80
KLFIGMVSK	O95319	0.80
GGLVNYFK	O95721	0.80
SRMQSTYNY	O95793	0.80
LPSSGRSSL	O95817	0.80
LPSLRILYM	P00403	0.80
AQNSVIIVDK	P02786	0.80
HPDYGSHIQAL	P04040	0.80
SPQKVTLYL	P05107	0.80
IPKSAVGEL	P05107	0.80
GLFGGAGVGK	P06576	0.80
SVYYNEATGGK	P07437	0.80
NPQERTLTL	P08238	0.80
WEQKATVI	P08575	0.80
ALRPSTSRSLY	P08670	0.80
TLLGFFLAK	P09884	0.80
IFDLGGGTF	P11142	0.80
ILNVSAVDK	P11142	0.80
AVYTKMMTKK	P11413	0.80
YTKMMTK	P11413	0.80
AVYTKMMTK	P11413	0.80
RLVQGSILKK	P12004	0.80
TPLSSTVTL	P12004	0.80
HVSLVQLTL	P12004	0.80

QSFDKEWVAK	P13073	0.80
VPRASVPDGFL	P14174	0.80
SFIAYQKK	P14209	0.80
MPLEDMNEF	P15927	0.80
QPRYPVNSV	P17987	0.80
YTDSYTDTY	P18583	0.80
PKFEVIEKPQA	P18859	0.80
KFEDPKFEVIEKPQA	P18859	0.80
VLFAGQHIAK	P21333	0.80
RIATGSFLK	P22234	0.80
RVFVVGVGMTK	P22307	0.80
FPNAIEHTL	P22314	0.80
FPNAIEHTL	P22314	0.80
FPNAIEHTLQW	P22314	0.80
FPNAIEHTLQW	P22314	0.80
MPLGSAVDIL	P25205	0.80
ILANDGVLL	P25789	0.80
ILFPRKPSAPK	P26373	0.80
TAVKNDYEM	P27694	0.80
QLIAKDDQLK	P27708	0.80
LPTEKEVAL	P27816	0.80
GTLSGWILSK	P27824	0.80
VPRAAFLSPLLPL	P30040	0.80
KVVEGSFVYK	P31150	0.80
SIFGGTDMKK	P33992	0.80
APRSQYTTGRGSSGVGL	P33993	0.80
RLMNETTAVALAY	P34932	0.80
ENYPTY	P35659	0.80
IPEEILGKVSI	P36507	0.80
KTKEAVLLLK	P36578	0.80
FRIDGPVISESTP	P42167	0.80
AIALALREK	P42285	0.80
AIALALREK	P42285	0.80
DVLCKLVEKGE	P42704	0.80
RPQTGLSFL	P43686	0.80
EPIPYEFMA	P46778	0.80
RPSGPSKAL	P48444	0.80
HPLGMAIFL	P49327	0.80
HPLGMAIFL	P49327	0.80
APLDSIHSL	P49327	0.80
APLDSIHSL	P49327	0.80
LLDPQLVPGG	P49368	0.80
RLLTSLRAK	P49368	0.80
MLREQKEQLQK	P49750	0.80
MPPPPGPAL	P49750	0.80
KVLPLLKIIKK	P49792	0.80
KVFGGTVHK	P49915	0.80
YYPTSSSTSF	P50402	0.80
TPDGIDLRL	P50416	0.80
RVDQIIMAK	P50990	0.80
RIYGESADAVK	P51114	0.80
GLFEVGAGWIGK	P51659	0.80
RMGPVMDRMATGLE	P52272	0.80
RMGPVMDR	P52272	0.80
FISLL	P52292	0.80
YRPETGSWVF	P52948	0.80
SLVGGLLQSK	P52948	0.80
AIYLVTSLASK	P55060	0.80
DVFERPSAKK	P55081	0.80
SPEAQPKTL	P55265	0.80
QPEGMISESL	P55265	0.80

MKMPTMVPL	P56270	0.80
FPEHIFPAL	P61160	0.80
ITTQVTI	P61978	0.80
ITTQVTI	P61978	0.80
RILSGVVTK	P62280	0.80
TAMDVVYAL	P62805	0.80
YVLGYKQTLK	P62888	0.80
SPNKLYTL	P62899	0.80
STIKFQMKK	P62906	0.80
YIKSTMGKPQRLY	P62906	0.80
APAGRKVGL	P62917	0.80
ATDPNILGR	P78527	0.80
SVKVYF	Q00325	0.80
RQIPYTMMK	Q00325	0.80
HPESERISM	Q01082	0.80
IPYHSEVPVSL	Q01082	0.80
HPDQAQAIL	Q01082	0.80
EIQQEM	Q01826	0.80
RVMQIIVKGK	Q04759	0.80
KFDLSRATF	Q04759	0.80
MPDGQFKDISL	Q06830	0.80
STFNQVVLK	Q07020	0.80
RLYLNVLNR	Q10713	0.80
RPMGAGEAL	Q12906	0.80
WRAPIASIHSF	Q13015	0.80
AEGDLIEHF	Q13151	0.80
VLAPEGSVANK	Q13283	0.80
LPRAVGTQTL	Q13596	0.80
STLPKSLLK	Q14008	0.80
SYRDSYDSY	Q14011	0.80
TYDLQESNV	Q14141	0.80
AVADKVHLMYK	Q14258	0.80
AVYQARPQLHK	Q14258	0.80
GDILVF	Q14562	0.80
NPRFGGKEL	Q14566	0.80
SELLPAK	Q14980	0.80
HPFPESKPVL	Q15005	0.80
KVNIVPVIAK	Q15019	0.80
SPQGRVMTI	Q15365	0.80
SPISSDQSL	Q15648	0.80
APKAGPGVV	Q15691	0.80
KVNIIPLIAK	Q16181	0.80
KTYEVSLREK	Q16531	0.80
AVYSMVEFNGK	Q16531	0.80
TRMQNDSILK	Q5JTV8	0.80
VSAAAEF	Q5SW79	0.80
FPVSKTLVL	Q5UIP0	0.80
RSIPQSATWK	Q86YP4	0.80
RPRPDEERPL	Q86YV0	0.80
MPALRSINL	Q8TCA0	0.80
RVYGGLTTK	Q8WUM4	0.80
VVYGGKSTIR	Q8WWY3	0.80
ELNPSIPLL	Q8WXH0	0.80
SPHDISNVL	Q92619	0.80
YSQNQQSY	Q92804	0.80
RVQEAVESMVK	Q96C01	0.80
RLSQLKQLLK	Q96CT7	0.80
AQYQFTGIKK	Q96IX5	0.80
MPESEEIAQLL	Q96JB5	0.80
RIFDLGRKK	Q96L21	0.80
SPKPPTSMF	Q99081	0.80

GMANLLTGPK	Q99536	0.80
VPKYWGSGL	Q99986	0.80
VTAVPTLLK	Q9BRA2	0.80
VRTPYTMSY	Q9BWF3	0.80
LTEQYNEQY	Q9BWF3	0.80
NPKRQTLVF	Q9GZR7	0.80
YYVNRDTL	Q9H0A0	0.80
IPLPLVKSL	Q9H0A0	0.80
SLMHSFILK	Q9NP97	0.80
NIAIEF	Q9NTJ3	0.80
SPSSVTGNAL	Q9NYB0	0.80
NTEEEGLKY	Q9NYF8	0.80
LSVSLL	Q9NYH9	0.80
KPKAPPPSL	Q9NZZ3	0.80
SPRGFPLGL	Q9P270	0.80
TPVDDRISL	Q9UHI6	0.80
LFDLGGQYL	Q9UHX1	0.80
GLWHMKTYK	Q9ULC4	0.80
LQIQQGLQTL	Q9UMX0	0.80
VLAPEGSVPNK	Q9UN86	0.80
ARPPPSMSA	Q9UQ35	0.80
FLDLTEQEF	Q9Y262	0.80
LPDSGHLHPL	Q9Y285	0.80
SLVGQTSPK	Q9Y2X3	0.80
TTLGKELASK	Q9Y3D8	0.80
FPSAPDVKA	Q9Y3F4	0.80
TSHLF	Q9Y520	0.80
TSHLF	Q9Y520	0.80
LPSASHFSQL	Q9Y520	0.80
LPSASHFSQL	Q9Y520	0.80
KTKELIGSMK	Q9Y5J1	0.80
RIVSAQSLAEDDVE	Q15388	0.95
RIFTLKPVRK	Q96EE3	0.95
FPVEVNTVL	O14744	1.00
MPEPDAQRF	O14757	1.00
KLIDIVSSQK	O14757	1.00
VPMFRNVSL	O14980	1.00
VVFKLLGPVLVK	O15212	1.00
LLGPVLVK	O15212	1.00
RLFVGSIPK	O43390	1.00
RLFVGSIPK	O60506	1.00
YPDRIMNTF	P07437	1.00
SLPLVDTHSK	P08670	1.00
YPEEVSSMVL	P11142	1.00
NINGQIPTGEGPPLVK	P11171	1.00
KTWTVVDAKTLKK	P13010	1.00
SFIAYQK	P14209	1.00
LPFGKVTNL	P26599	1.00
KAFNQGKIFK	P26641	1.00
RIFQEPTEPK	P31350	1.00
SYGGQQQSY	P35637	1.00
NRPGTVSSL	P35659	1.00
KVFDGIPPPY	P40429	1.00
RSDTPLIYK	P41252	1.00
HPLHNDPNL	P42285	1.00
HPLHNDPNL	P42285	1.00
VIDEPVRL	P46013	1.00
GDKEPGLPP	P48634	1.00
TFDDIVHSF	P49327	1.00
TFDDIVHSF	P49327	1.00
HPEPASGLAAL	P49327	1.00

HPEPASGLAAL	P49327	1.00
LEGSGLESII	P49327	1.00
LEGSGLESII	P49327	1.00
SPQAPTHFL	P49773	1.00
HPTIISESF	P50991	1.00
YPFTGDHKQKF	P53396	1.00
VFDEAIRAVL	P60763	1.00
SSGPERIL	P61978	1.00
SSGPERIL	P61978	1.00
RPLSKTVRF	P62280	1.00
KVAPAPAVVK	P62424	1.00
RLVTPRVLQHK	P62753	1.00
RPFPGLVI	P78347	1.00
LKVKGNVFK	P84098	1.00
IVDGNHRLTL	Q01082	1.00
KIDMPHRFK	Q04759	1.00
APASPFRQL	Q07021	1.00
NPRLNILDTL	Q13200	1.00
RPADKRSFIP	Q14137	1.00
TIYTSYKEK	Q15005	1.00
GTYVKPLSNK	Q15067	1.00
RLYEWTTEK	Q16531	1.00
QVLNADAIVVK	Q7KZF4	1.00
LPSKVPTTL	Q7Z434	1.00
APPRPGSSF	Q86XP3	1.00
APRAPAAGL	Q86YV0	1.00
APRAGPGQL	Q86YV0	1.00
AQYGNILKH	Q8NFH5	1.00
LPAEFFEVL	Q8TEM1	1.00
SGVPAL	Q8WWM7	1.00
RVFLRAINK	Q92608	1.00
TTDSSYGQNY	Q92804	1.00
MRLSQL	Q96CT7	1.00
SPQSPGDAL	Q96KR1	1.00
YRAQPSASL	Q96PK6	1.00
KLLDVVHPAAK	Q99832	1.00
SPAVERLISSL	Q9BQ52	1.00
ALKTGIVAK	Q9BQ61	1.00
KLFNPPEESEK	Q9NRP2	1.00
AVNPKEIASK	Q9NTJ3	1.00
AELKSL	Q9NTJ3	1.00
AVRTLNKVAMK	Q9UBF2	1.00
